# Increased compensatory kidney workload results in cellular damage in a short time porcine model of mixed acidemia – Is acidemia a ‘first hit’ in acute kidney injury?

**DOI:** 10.1371/journal.pone.0218308

**Published:** 2019-06-17

**Authors:** Martin Russ, Sascha Ott, Janis R. Bedarf, Michael Kirschfink, Bernhard Hiebl, Juliane K. Unger

**Affiliations:** 1 Department of Anesthesiology and Operative Intensive Care Medicine, Campus Virchow-Klinikum and Campus Charité Mitte, Charité–Universitätsmedizin Berlin, Berlin, Germany; 2 Department of Experimental Medicine, Campus Virchow-Klinikum, Charité–Universitätsmedizin Berlin, Berlin, Germany; 3 Department of Neurology, University of Bonn, Bonn, Germany; 4 German Centre for Neurodegenerative Disease Research (DZNE), Bonn, Germany; 5 Institute for Clinical Immunology, University Hospital Heidelberg, Heidelberg, Germany; 6 Institute for Animal Hygiene, Animal Welfare and Farm Animal Behaviour and Virtual Center for Replacement–Complementary Methods to Animal Testing, University of Veterinary Medicine Hannover, Foundation, Hannover, Germany; The University of Manchester, UNITED KINGDOM

## Abstract

Acute kidney injury (AKI) corrupts the outcome of about 50% of all critically ill patients. We investigated the possible contribution of the pathology acidemia on the development of AKI. Pigs were exposed to acidemia, acidemia plus hypoxemia or a normal acid-base balance in an experimental setup, which included mechanical ventilation and renal replacement therapy to facilitate biotrauma caused by extracorporeal therapies. Interestingly, extensive histomorphological changes like a tubular loss of cell barriers occurred in the kidneys after just 5 hours exposure to acidemia. The additional exposure to hypoxemia aggravated these findings. These ‘early’ microscopic pathologies opposed intra vitam data of kidney function. They did not mirror cellular or systemic patterns of proinflammatory molecules (like TNF-α or IL 18) nor were they detectable by new, sensitive markers of AKI like Neutrophil gelatinase-associated lipocalin. Instead, the data suggest that the increased renal proton excretion during acidemia could be an ‘early’ first hit in the multifactorial pathogenesis of AKI.

## Introduction

Acute kidney injury (AKI) complicates the course of disease of up to 50% of all patients admitted to an intensive care unit [1–3] and contributes to the failure of further organs, especially the lungs [4]. This ‘kidney-lung cross-talk’ seems to take place bidirectional [5–9] and the pathophysiological mechanisms include the release of proinflammatory mediators due to mechanical ventilation, impaired circulation due to positive end-expiratory airway pressure (PEEP), as well as hypercapnia and hypoxia due to acute lung injury (ALI) or lung protective low tidal volume ventilation (LTVV) [10]. The implementation of LTVV and adequate PEEP levels reduce the mortality due to ALI [11, 12], but the combination of lung injury and LTVV can cause respiratory acidosis. In patients, respiratory acidosis is usually tolerated to facilitate lung protective ventilation regimes as long as arterial pH remains above 7.2; this approach is called ‘permissive hypercapnia’. [13, 14].

Elimination of a CO_2_ through the lungs and elimination of nonvolatile acids through the kidneys are the main regulators of acid-base balance. The kidneys have to increase acid elimination and retention of bicarbonate to protect pH homeostasis if the respiratory control of CO_2_ is impaired. The renal excretion of hydrogen ions is an active, energy consuming process that depends H^+^-ATPases, which exist ubiquitously in the nephrons with the highest concentration in intercalated cells [15, 16]. Still, there is little knowledge whether the ‘additional workload’, which is forced on the kidneys in case of impaired CO_2_-removal through the lungs, contributes to AKI. Furthermore, a reduction of mainly oxygen-dependent ATP-generation–as it can occur in ALI–might aggravate AKI further. Therefore, the hypothesis, that a short period of acidemia as well as a short period of combined acidemia and hypoxemia is an independent risk factor for the development of AKI, was tested. The investigations were performed in an experimental porcine model that enabled ‘standardized’ acidemic/hypoxemic conditions for several hours and a fast correction of the induced pathology within one hour before sacrificing the animals for kidney biopsies. The model accounted for the acute biocompatibility reactions of critically ill patients to ‘common’ organ support therapies like mechanical ventilation and renal replacement therapy.

## Materials and methods

### Animal wellfare

The animals were held under standard conditions at the Department of Experimental Medicine (facilities are certified by ISO 9001:2000) according the guidelines for laboratory animal care of the European and German Societies of Laboratory Animal Sciences. The study protocol was approved by the Charité veterinarian and the University Animal Wellfare Officer as well as the federal authorities for animal research in Berlin (LaGeSo, Landesamt für Gesundheit und Soziales, approval number G 0380/05), Germany.

### General study design and study groups

All experiments were performed in male pigs (German landrace, large white, body weight of 37–42 kg, age of 3–4 months). Pigs were assigned to either of 4 groups with 6 animals per group (n = 6): mixed acidemia with continuous veno-venous hemofiltration (CVVH) or without (control) as well as mixed acidemia plus hypoxemia with CVVH and without (control). A full description of all used materials and methods is included in the supplementary materials. Two further groups with CVVH (n = 6) and without control, n = 5) were exposed to normoxemia and a physiological acid-base balance. Due to ethical aspects and strict local animal protection laws the normoxemic control group with normal acid-base balance is a control group of a former study (control group from Unger et al. [17]). Thus, only qualitative comparisons without statistical tests were performed between the normoxemic control-group with normal acid-base balance and the other study groups.

### Anesthesia and instrumentation

General anaesthesia was induced in pigs after premedication, followed by orotracheal intubation and continuous intravenous anaesthesia (S1 Table). Initially, the pigs were normoventilated in with an end-tidal CO_2_ (etCO_2_) target of 35–40 mmHg and a saturation of peripheral oxygen (SpO_2_) above 95%. After intubation the animals were placed in a supine position and cannulated with an arterial line, central venous line, shaldon catheter for CVVH and pulmonary arterial catheter (PAK). The urinary bladder was directly catheterized with a balloon catheter.

### Acidemia and hypoxemia protocol and CVVH

**Fig 1:**
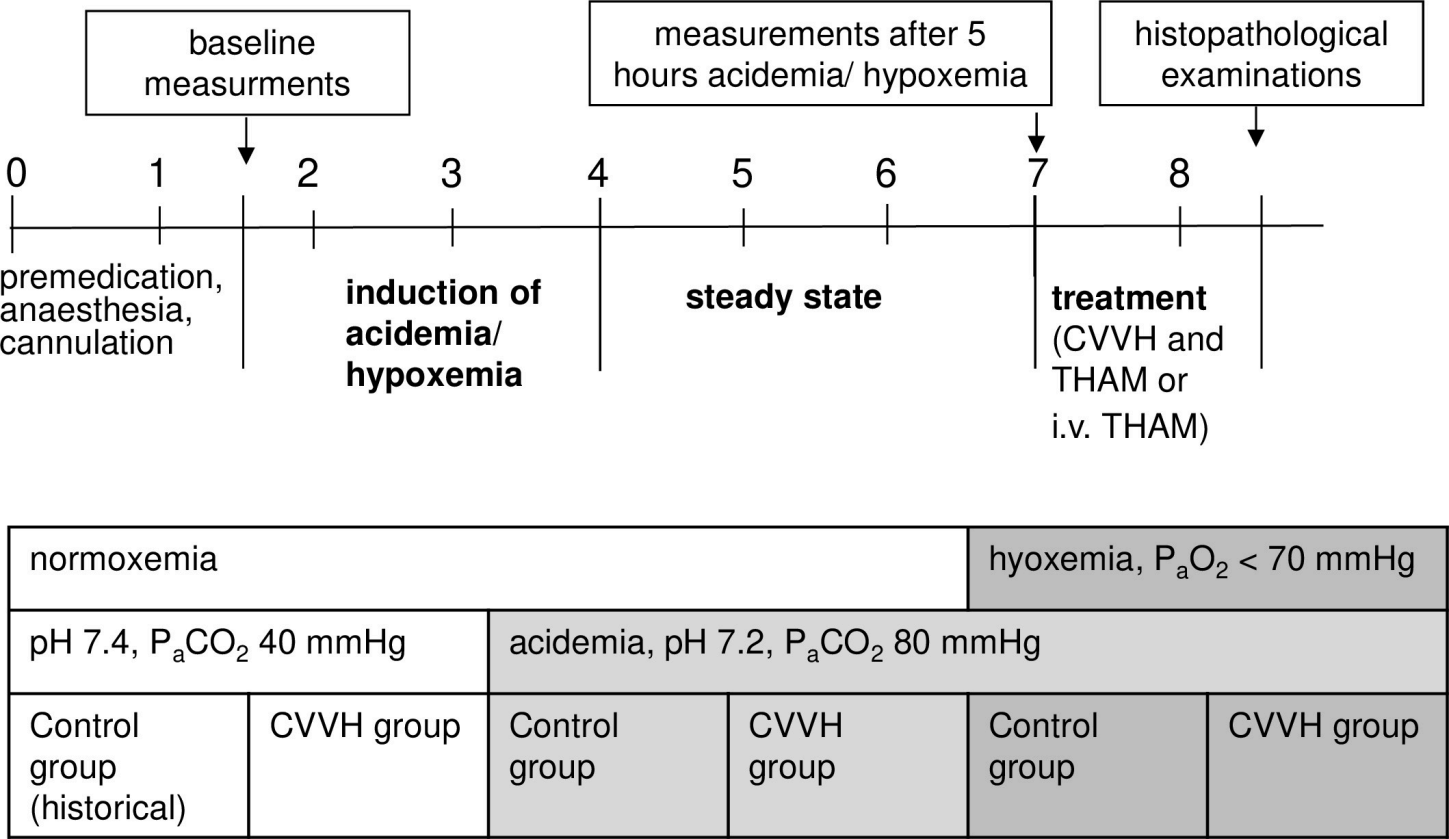
Schematic draw of the experimental protocol and experimental groups.

After baseline measurements, acidemia or acidemia/hypoxemia was induced (Fig 1). Mixed acidemia was achieved by infusion of an acid solution (0.2 M lactic acid and 0.2 M hyperchloremic acid diluted in 0.9% saline) and low tidal volume ventilation (6 ml/kg; arterial pH 7.19–7.24; P_a_CO_2_ of 80–85 mmHg). Hypoxemia was induced by reduction of F_I_O_2_ until P_a_O_2_ was below 70 mmHg and S_v_O_2_ below 65%. The acid infusion rate had to be adjusted individually in the hypoxemia-groups to reach the target range, since hypoxemia induced a metabolic acidosis itself (S1 Table).

**Fig 2:**
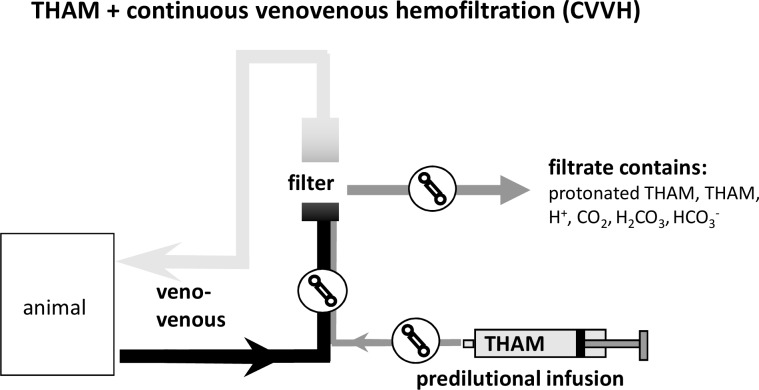
THAM-infusion and CVVH. The drawing illustrates the buffer properties of predilutional (before the hemofilter) infusion of tris-hydroxymethylaminomethane (THAM) in the hemofiltration circuit.

Then, respective pigs were connected to the CVVH which was operated for three hours by recirculation of the filtrate into the venous bubble trap while acidemia or acidemia/hypoxemia was maintained (total exposure to acidemia or acidemia/hypoxemia for five hours.; Fig 1). After three hours of stable acidemia or acidemia/hypoxemia the disorder was corrected with predilutional (before the hemofilter; Fig 2) infusion of 8 mmol/kg/hr of tris-hydroxymethylaminomethane-buffer (THAM) in the CVVH-groups or central-venous infusion of THAM (2mmol/kg/hr) in the control groups. CVVH-groups and control-groups received a different dosage of THAM due to the partial immediate filtration of THAM in the CVVH-animals. Hypoxemia was corrected during this ‘treatment’ period by adjusting F_I_O_2_ to 100%. Thus, final histopathology was performed after correction of mixed acidemia and hypoxemia.

### Laboratory analyses and hemodynamic measurements

All samples for blood analyses were drawn from the central venous catheter (or PAK for SvO_2_) and immediately processed. Blood samples were analysed at the local Institute for Clinical Chemistry, Charité—Universitätsmedizin Berlin using laboratory standard methods except for blood gas samples (ABL700; Radiometer, Copenhagen, Denmark measuring pH, P_a_O_2_, P_a_CO_2_, electrolytes: Na^+^, K^+^, Cl^-^, ionized Ca^2+^ and lactate). Serum and urine levels of IL-18, IL-6 and TNF-α were determined by commercially available enzyme linked immunoabsorbant assay (ELISA) (S7 Table).

Cardiac output (CO) was measured with the thermodilution technique and pulmonary artery occlusion pressure (PAOP) as determinant for left ventricular preload was measured at all main time points of the experiment using the PAK. Furthermore, oxygen delivery (DO_2_) and oxygen consumption (VO_2_) were calculated using standard equations [18].

### Histopathology and score criteria

After the experiment the animals were euthanized with an overdose of fentanyl (0.5 mg), thiopental (1 g) and potassium chloride (60 mval), the kidneys were immediately removed and small corticomedular tissue samples were fixated in a 4 vol% buffered formalin solution. The no-flow time of the kidneys was about ten minutes. The tissue samples were embedded in paraffin; slides of 4 μm thickness were cut and stained with standard hematoxylin and eosin (HE)-staining. Kidney cell damage was analysed with a semiquantitative scoring system by adjusting existing scores [19–21] to our investigation (S2 Table and S3 Table). Tissue samples were evaluated at a 400:1 field enlargement. Multiple visual fields were scored for both kidneys of each animal (median value of 10 visual fields of each kidney equals one score value).

### Immunohistochemistry (IHC) staining and ELISA analysis

IHC staining was performed for IL-18, IL-6 and TNF-α with an ABC-staining kit in concordance with the producer’s standard protocol (supplementary materials and S4–S6 Tables).

### Statistical analysis

All data were analysed using Sigma Stat 3.1 for Windows. Non-parametrical tests were used, because some data failed normal distribution. Kruskal-Wallis One Way Analysis of Variance on Ranks was used to test for possible differences of the baseline values between groups. The intergroup comparison between two respective study groups (e.g. academic control vs. control group with normal acid base balance) was performed using the Mann-Whitney Rank Sum Test. Post hoc analysis (pairwise comparison) was done using either Tukey’s test for equal sample size or Dunn's method for an unequal sample size. Friedman Repeated Measures Analysis of Variance on Ranks was performed for intra-group comparisons. Statistical significance was assumed at p <0.05.

## Results

### Mechanical ventilation and systemic mediator release–ventilation alone did not cause a relevant biotrauma

Plasma IL-6 levels increased in tendency in all groups during the experiment, except in the normoventilated control-group, which was exposed to the highest inspiratory pressures (albeit only 22 mmHg, Table 1). A significant increase in plasma IL-6 levels was observed in the acidemic/hypoxemic CVVH-group, which was presumably exposed to the highest biotrauma with respect to compensating low pH values under hypoxemic conditions and simultaneous biocompatibility reactions to the CVVH circuit (Table 1). The same pattern was observed for plasma levels of IL-18 (Table 1).

**Table 1:** Mediator levels, hemodynamic, renal and blood gas parameters.

	pH 7.4				pH 7.2 (mixed acidemia)							
	normoxemia								hypoxemia			
	control		CVVH		control		CVVH		control		CVVH	
	baseline	a. 5h	baseline	a. 5h	baseline	a. 5h	baseline	a. 5h	baseline	a. 5h	baseline	a. 5h
**1.1 Mechanical ventilation and markers for biotrauma**												
Peak inspiratory pressure [mbar]	22 (22/24)	22 (21/24)	21 (20/22) ‡	21 (20/22) #	18 (18/27)	14 (14/19)	18 (16/20) ‡	17 (16/20) #	20 (20/20)	20.0 (18/20)	20 (20/22)	18 (14/20)
pulmonary atelectasis	40–65%				10–25%							
IL-6 plasma [pg/ml]	11 (8/11)	11 (10/13)	0 (0/109)	93 (0/115)	107 (0/263)	274 (143/460)	0 (0/0)	93 (0/174)	0 (0/0)	77 (0/236)	0 (0)	97 (41/172)
TNF-α plasma [pg/ml]	0 (0/15)	9 (2/16)	37 (6/45)	44 (38/45)	30 (5/78)	56 (6/71)	47 (0/68) #	26 (12/35) §	69 (55/97)	14 (0/47)	117 (102/322)#	53 (39/77) §
IL-18 plasma [pg/ml]	509 (417/703)	406 (354/461)	12 (7/14)	80 (61/108) #	23 (7/214)	354 (134/ 627)	52 (42/72)	249 (162/357)#	273 (211/353)	373 (319/474)	244 (222/258)	465 (300/675)
**1.2 Hemodynamic parameters and oxygenation**												
PaO_2_ [mbar]	170 (160/191)	163 (122/211)	430 (394/472)	422 (387/456)	397 (257/429)	219(135/ 338)#	457 (436/478)	350 (287/399)§	412 (378/440)	79 (68/88) #,$	490 (467/496)	62 (48/64) §,$
SvO_2_ [%]	78 (68/84)	56 (55/69)	77 (75/80)	72 (66/74)	82 (84/89)	67 (53/72)	78 (78/80)	66 (64/68) †	79 (78/80)	60 (38/61)	80 (76/82)	28 (21/40) †
MAP [mmHg]	70 (65/81)	115 (108/115)	110 (99/115)	90 (72/97)	103 (98/103)	72 (67/77)	97 (93/104)	66 (64/68) §	88 (84/98)	57 (46/60)	117 (104/121)	49 (47/54) §
CVP [mmHg]	6 (4/8)	6 (3/7)	8 (7/9)	8 (7/9)	9 (7/11)	12 (10/14) ‡	5 (2/7)	6 (3/9) ‡	6 (3/10)	7 (5/13)	9 (7/11)	11 (9/12)
PAOP [mmHg]	6 (6/10)	8 (6/11)	10 (9/12)	10 (9/12)	9 (7/10)	11 (10/14)	8 (6/10)	10 (8/11)	10 (7/10)	13 (12/17)	10 (9/12)	10 (9/12)
CO [l/min]	5.3 (5.2/5.5)	4.1 (3.6/4.1)	4.2 (3.9/4.6)	3.4 (3.0/4.0)	4.7 (4.4/4.8)	3.5 (2.9/4.1)	3.9 (3.5/4.5)	3.5 (3.3/3.9)	4.0 (3.9/4.1)	4.0 (3.4/4.8)	3.7 (3.3/4.1)	3.9 (3.8/4.2)
SVR [dyn*sec*cm^-5^]	875 (838/1430)	2207 (1711/ 2625)	1880 (1724/ 2139)	1626 (1525/ 1688)	1589 (1361/ 1816)	1350 (1205/ 1652) ‡	1912 (1745/ 2254)	1282 (1217/ 1294) #	1543 (1466/ 1648)	892 (819/ 1059) ‡	2252 (2142/ 2293)	732 (592/ 879) #
DO_2_ [ml/min]	632 (586/717)	452 (440/500)	550 (467/613)	370 (341/432)	593 (499/655)	396 (339/505)	525 (483/555)	422(407/ 456)†	553 (500/620)	395 (341/515)	454 (401/501)	332 (263/364)†
VO_2_ [ml/min]	161 (89/169)	190 (146/216)	164 (154/173)	141 (118/164)	126 (114/141)	164 (156/212)	140 (112/152)	167 (163/169)	150 (132/172)	179 (175/185)	118 (114/125)	180 (178/186)
**1.3 Acid-base homeostasis and kidney function**												
pH arterial blood	7.47 (7.46/7.53)	7.51 (7.5/7.53)	7.48 (7.47/7.5)	7.44 (7.42/7.47)	7.44 (7.43/7.45)	7.25 (7.23/ 7.28) † §	7.45 (7.43/7.46)	7.22 (7.21/ 7.23) †	7.49 (7.47/7.5)	7.22 (7.21/7.25)	7.46 (7.45/ 7.48)	7.23 (7.23/7.24)
P_a_CO_2_ [mbar]	40 (39/42)	39 (38/39)	44 (44/45)	43 (42/46) #	46 (45/48)	80 (73/86)	47 (44/48)	84 (82/87) #	43 (41/44)	77 (73/78) §	45 (43/46)	92 (88/95) §
diuresis [ml/kg/h]	1.2 (0.3/3.4)	1.5 (1.4/1.7)	7.2 (6.4/18.0)	7.2 (5.6/13.6)	5.2 (2.6/7.8)	7.0 (4.6/10.4)	7.2 (5.2/12.4)	9.8 (8.4/17.0) §	10.0 (5.8/12.6)	3.8 (1.4/5.2)	6.6 (5.2/8.4)	3.4 (1.0/5.8) §
creatinine clearance [ml/min]	32.7 (20.9/ 118.8)	93.9 (80.1/ 115.8)	42.5 (18.3/ 114.1)	70.9 (64.3/ 110.1)	52.3 (47.1/81.5)	61.4 (48.1/ 83.9)	52.2 (27.3/ 121.2)	48.9 (7.5/66.1)$	64.7 (57.1/ 172.1)	80.1 (58.4/90.7) $	45.8 (32.0/ 85.9)	27.8 (15.3/76.1)
pH urine	7.83	7.88	7.4 (7.1/7.7) $ ß	7.25 (7.2/7.4) +Δ	6.95 (6.0/7.1) $	6.2 (6.0/6.5) Δ	6.75 (5.9/7.1) ß	6.2 (5.7/6.8) +	6.85 (6.1/7.3)	6.15 (6.0/6.2)	6.75 (6.4/7.0)	6.65 (6.5/6.7)
IL-18 urine [pg/ml]	177 (148/268)	239 (214/311)	136 (111/178)	96 (85/102)	109 (98/123)	109 (80/134)	119 (105/134)§	81 (69/115)	119 (111/134)$	115 (104/117)	65 (62/94) § $	71 (61/76)
NGAL urine [RU*100/ml/kg/h]	1418.8 (326.4/ 1745.6)	282.4 (194.0/ 422.0)	12.0 (4.6/ 20.0)	20.0 (4.2/29.2)	40.6 (27.6/ 105.4) §	34.0 (18.2/ 38.2)	12.0 (10.4/ 12.6) §	18.6 (10.6/ 20.2) $	18.6 (6.2/31.8)	98.4 (72.2/ 134.8)	27.8 (10.0/ 45.4)	77.6 (44.6/ 228.8) $
Serum creatinine [mg/dl]	1.2 (1.0/1.2)	1.2 (1.0/1.3)	1.0 (0.9/1.2)	1.2 (1.0/1.3)	1.0 (1.0/1.0)	1.2 (1.0/1.4)	1.1 (1.1/1.2)	1.1 (1.1/1.4)	1.2 (1.1/1.3)	1.4 (1.4/1.6)	1.1 (1.0/1.2)	1.5 (1.4/1.8)

Data are presented as median with 25th and 75th percentiles at baseline (after complete instrumentation of the animals) and after five hours exposure respective to the experimental protocol. Pairs of symbols mark (e.g. ††) significant difference between two groups, grey background marks significant difference between baseline value and value after 5 hours exposure (p < 0.05 for all). CVVH means continuous veno-venous hemofiltration, control means control groups (without CVVH) as described in the article. The groups are further divided into groups with ‘normal’ acid-base balance (pH 7.4), groups exposed to acidemia (pH 7.2) and additional hypoxemia (as described in the article).

### Mechanical ventilation, hemodynamics and kidney perfusion–moderate positive continuous airway pressure alone did not compromise diuresis

**Fig 3:**
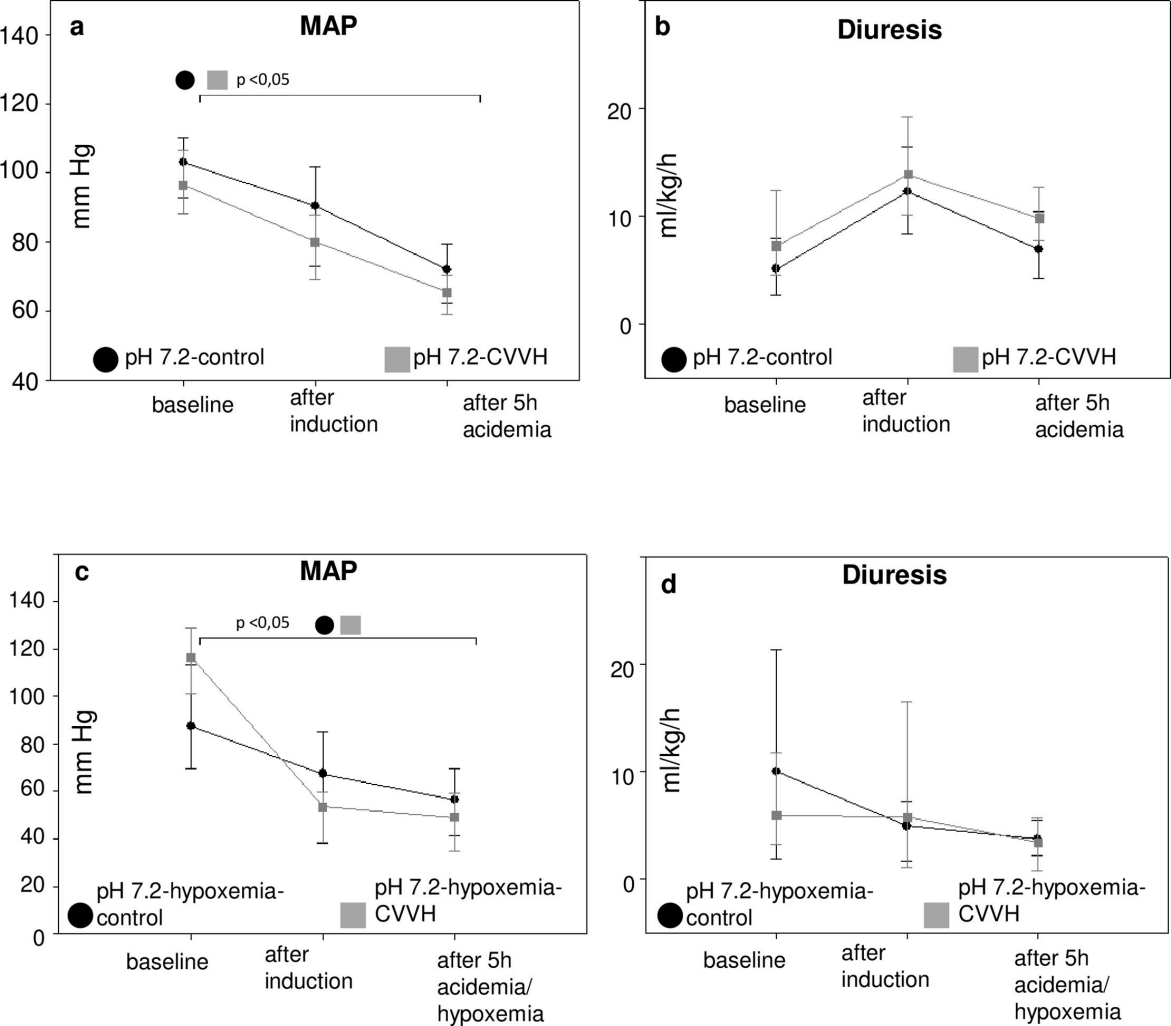
Mean arterial pressure and diuresis. Mean arterial pressures (MAP) and diuresis are depicted as line and scatter plots (median/25th/75th percentile) for animals exposed to acidemia (3.a, b) and acidemia with additional hypoxemia (3.c,d). The abbreviations pH 7.4-control. etc. mark the experimental groups as described in the article. Bracelets mark p < 0.0.5.

Mean arterial pressure (MAP) values decreased during the induction of acidemia, but remained above 65 mmHg. A moderate PEEP of 5 mmHg did most likely not result in a compromised kidney perfusion, since diuresis remained above clinically accepted cut offs for oliguria (0.5 ml/kg/hr) (Table 1, Fig 3). Additionally, creatinine clearance did not decrease significantly in any group and the use of advanced hemodynamic monitoring did not indicate a compromised systemic perfusion. As a result, it is unlikely that histomorphological kidney injury was caused by a compromised renal perfusion.

### Histopathology–short exposure to acidemia leads to cellular damage

**Fig 4:**
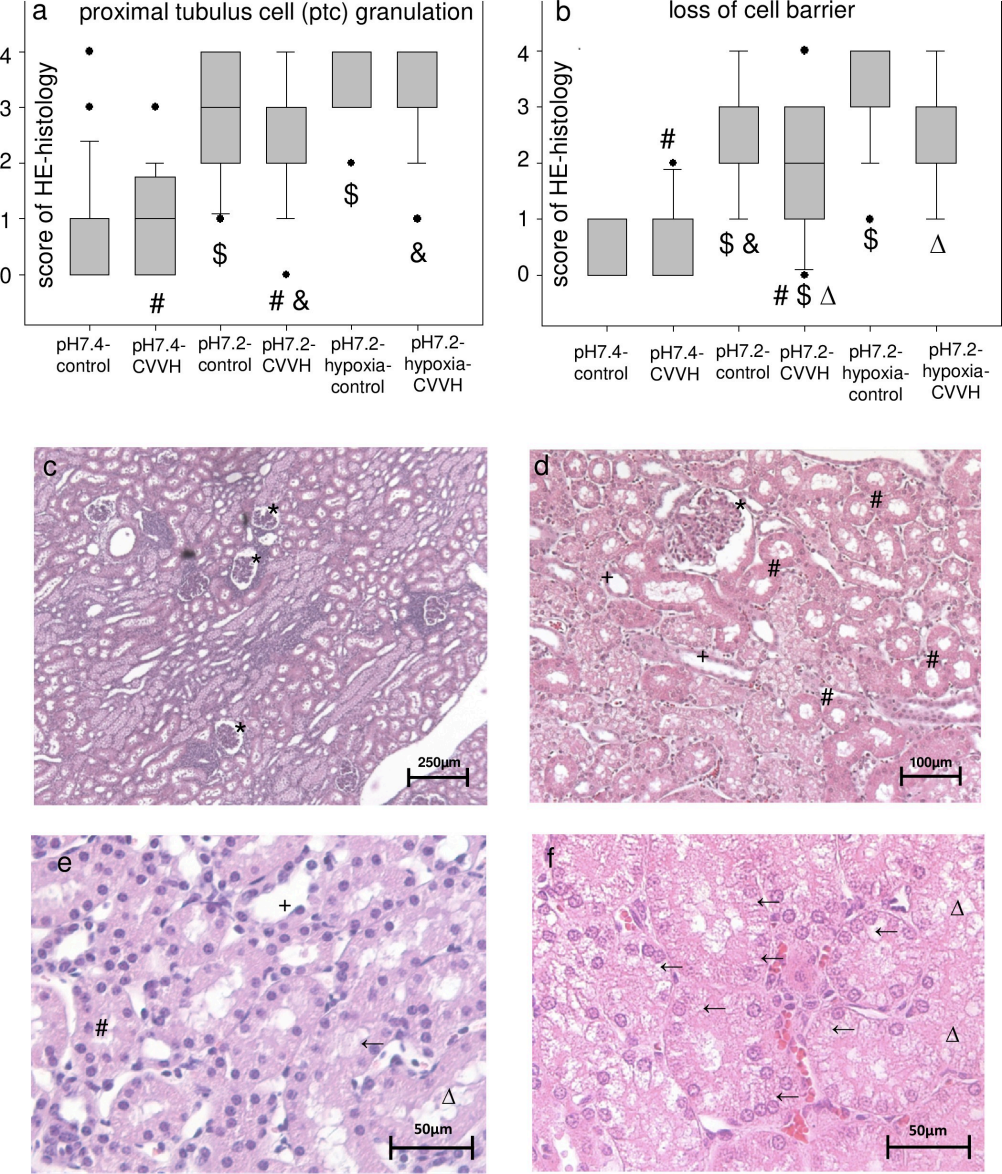
Short time acidemia results in kidney cell damage. Boxplots (median/25th/75th percentile/ outliers) (a, b) present the semiquantitative scores of the respective criteria for kidney cell damage. Y-axis: score from 0 (no damage visible) to 4 (damage in > 50% of the visual field) for the respective criteria. X-axis: study groups. Pairs of symbols mark p < 0.05. Photos (c, d, e, f) display typical kidney slices stained in hematoxylin eosin (HE, c. 50:1 enlargement, d. 200:1 enlargement, e, f at 400:1 enlargement). Graph e displays a slice with little kidney cell damage, whereas f is an exemplary photo of high kidney cell damage. * mark renal corpuscles, # mark proximal tubules, + mark distal tubules, ← mark proximal tubulus cell granulation, Δ mark loss of cell barrier.

Slices of kidneys tissue samples were scored using a semiquantitative score in HE staining (Fig 4, S3 Fig; score details are described in the supplemental methods). Scoring for histopathological criteria of kidney cell damage like granulation of proximal tubular cells (ptc granulation) resulted in higher score values in the groups exposed to acidemia with highest values in the acidemic/hypoxemic groups (Fig 4), despite the short period of exposure (five hours with induction) and correction of acidemia/hypoxemia before the end of the experiments. Notably, no significant differences were detected between the respective CVVH and control-groups, which suggests that the additional extracorporeal organ support system was not a trigger for kidney cell damage. Thus, the data indicate that even a short exposure to acidemia and acidemia/hypoxemia can result in kidney cell damage.

### Immunohistochemistry and plasma mediator concentrations–kidney cell damage was not caused by inflammation

**Fig 5:**
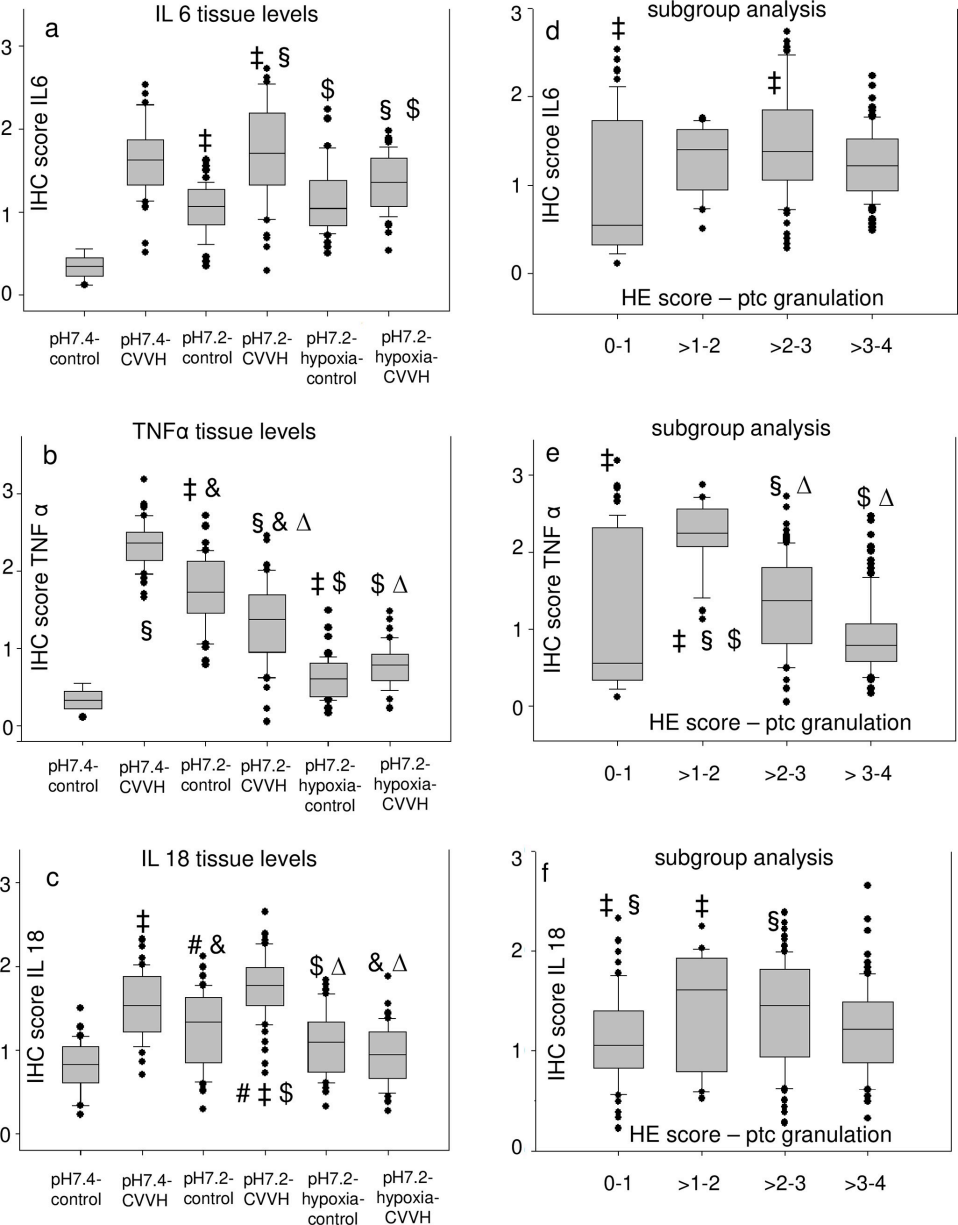
Kidney cell damage is not accompanied by high tissue levels of proinflammatory molecules. Boxplots present (median/25th/75th percentile/ outliers) the semiquantitative scores for immunohistochemical staining (IHC) (a, b, c) of kidney slices scored at 400:1 enlargement. X-axis: study groups. Pairs of symbols mark p < 0.05. The respective subgroup analysis (d, e, f) compares pooled subgroups for the semiquantative score for proximal tubular cell granulation (ptc) in hematoxylin eosin (HE) stained slices (0—no damage visible, 4 damage in > 50% of the visual field) with the highest/lowest scores for the respective immunohistochemical markers in immunohistochemical (IHC) stained slices.

**Fig 6:**
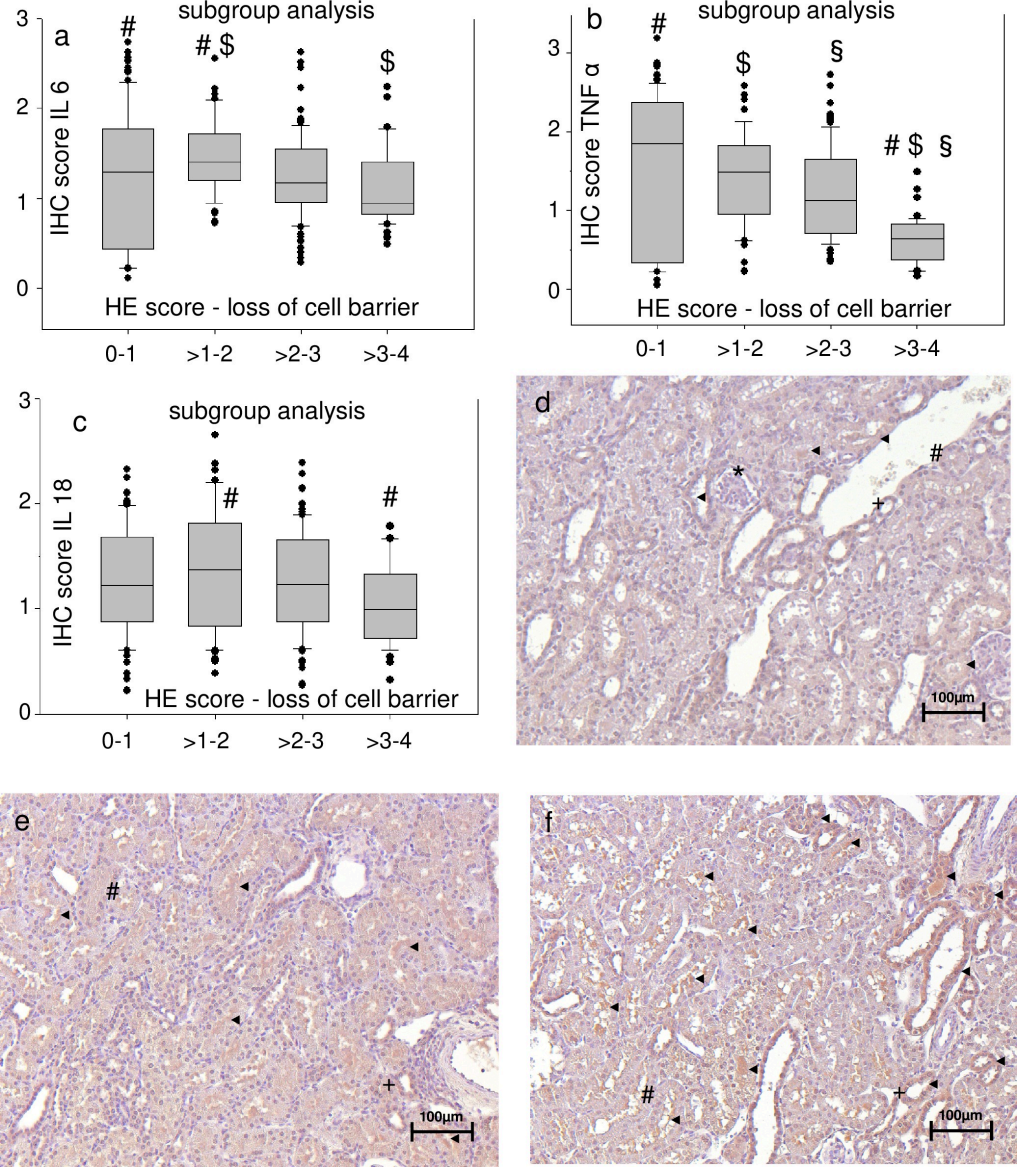
Kidney cell damage is not accompanied by high tissue levels of proinflammatory molecules independently of the score criteria that is investigated. Subgroup analysis (a, b, c) compares pooled subgroups for the semiquantitative score for loss of cell barrier in hematoxylin eosin (HE) stained slices with the highest/lowest scores for the respective immunohistochemical markers in immunohistochemical (IHC) stained slices at 400:1 enlargement. The values are presented as boxplots (median/25th/75th percentile/ outliers). Pairs of symbols mark p < 0.05. Photos (d, e, f) display kidney slices stained for IL 18 (all 200:1 enlargement) with d. little staining for IL 18 (IHC score 1), e. medium staining for IL 18 (IHC score 2), extensive staining for IL 18 (IHC score 3–4). * mark renal corpuscles, # mark proximal tubules, + mark distal tubules, ◄ mark staining for IL 18 (strained brown).

**Fig 7:**
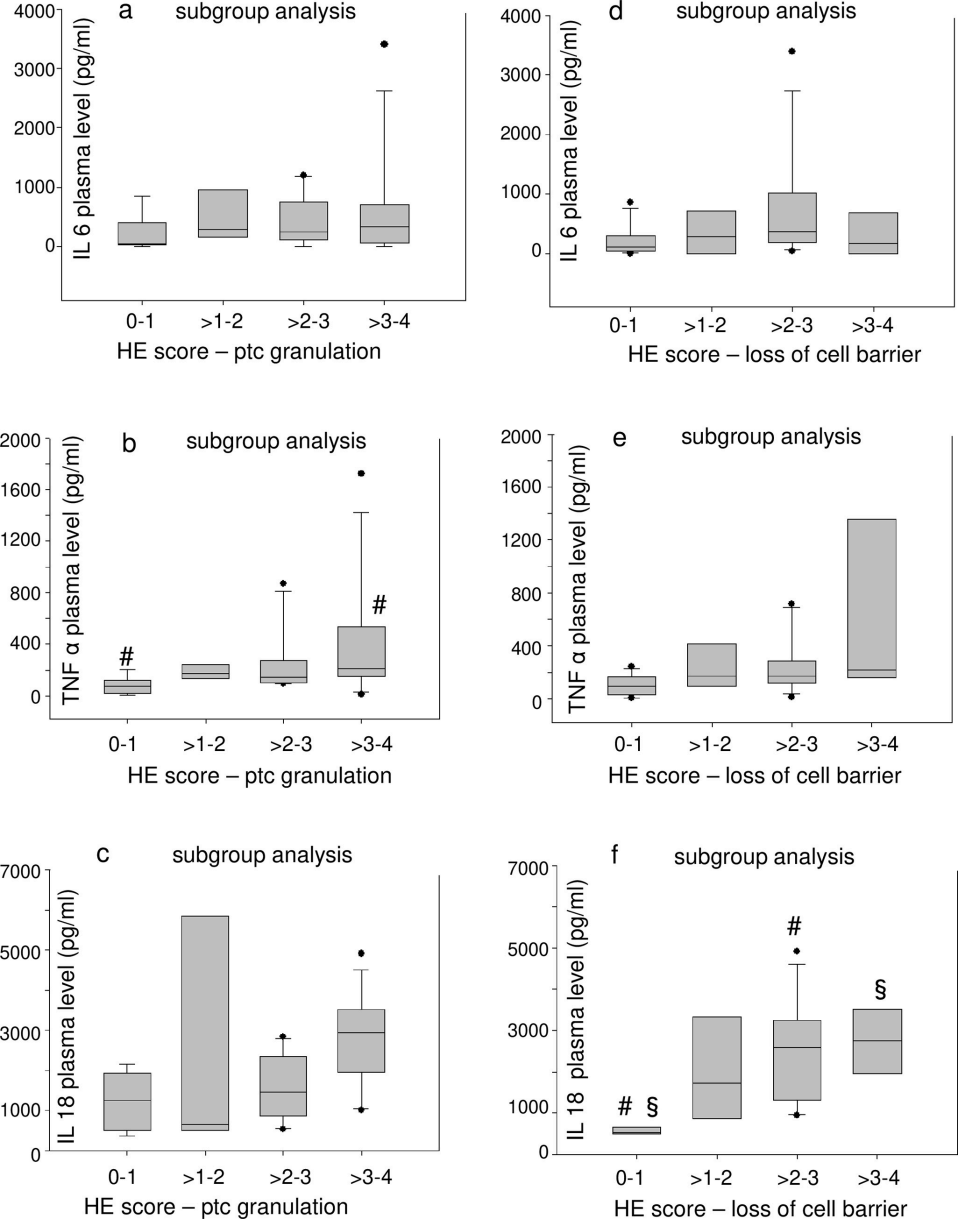
Kidney cell damage is not accompanied by high plasma levels of proinflammatory molecules. Subgroup analysis (a, b, c) compares pooled subgroups for the semiquantative score for proximal tubular cell granulation (ptc) in hematoxylin eosin (HE) stained slices at 400:1 enlargement with the plasma levels of proinflammatory mediators. A further subgroup analysis was performed using loss of cell barrier as criterion of cell damage (d, e, f). The values are presented as boxplots (median/25th/75th percentile/ outliers). Pairs of symbols mark p < 0.05.

Immunohistochemical (IHC) staining of kidney slices for IL-18, IL-6 and TNF-α (S3 Fig) was inconsistent compared to HE-staining. The groups with high HE-scores for cell damage–which were the groups exposed to acidemia/hypoxemia–did not have the highest scores for tissue levels of proinflammatory mediators (Fig 5). Pooled groups, which included all animals with the respective semiquantative HE-score within a range of two regardless of the experimental group (e.g. all animals with a score of 0–1 were pooled, all animals with a score >1–2, etc.), were formed for subgroup analysis. Interestingly, a pooling of animals with high HE-scores did not result in high tissue scores of proinflammatory mediators (Fig 5 and Fig 6). Consequently, the same test was performed for subgroups of HE scores for cell damage and plasma levels of proinflammatory mediators (Fig 7). Interestingly, pooled subgroups for high HE scores did have higher IL-18 (Fig 7) and TNF-α (Fig 7) plasma levels than groups with low HE scores, but this observation was not made for any other subgroup (Fig 7). In summary, the data does not indicate that kidney cell damage was triggered by local or systemic inflammation.

### Diuresis, acid-base homeostasis and kidney cell damage–kidney cell damage might have been triggered by acidemia

**Fig 8:**
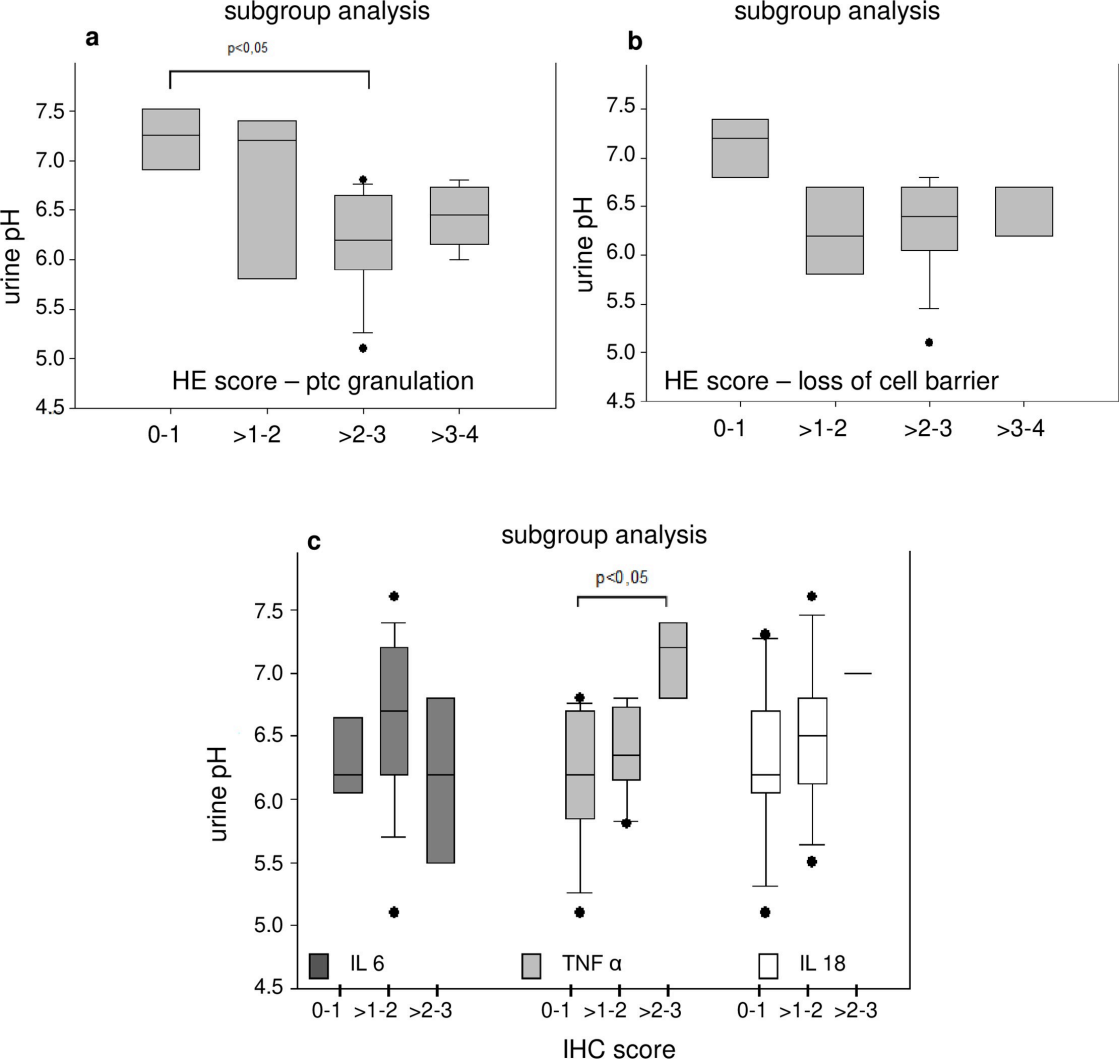
Histopathology as well as IHC scoring and urine pH. The respective subgroup analysis (a, b) compare pooled subgroups for the semiquantative score for proximal tubular cell (PTC) granulation (a) and loss of cell barrier (b) in hematoxylin eosin (HE) stained slices at 400:1 enlargement with urine pH values. Pooled subgroups for immunohistochemical (IHC) stained slices and urin pH are depicted in c. The values are presented as boxplots (median/25th/75th percentile/ outliers). Bracelets mark p < 0.05.

High diuresis rates were observed in all animals during acidemia/hypoxemia (3 ml/kg/hr) (Table 1). Subgroup analysis of pooled groups with high vs. low HE-scores for kidney cell damage and urine pH indicate that high HE-scores for cell damage were in tendency associated low urine pH values (not significant, Fig 8). These, findings were not matched by an analysis of pooled groups for high vs. low IHC-scores and urine pH (Fig 8). Again, the results do not support the hypothesis of an inflammatory response. Instead, the acid load was associated with early cellular kidney damage.

### Kidney cell damage was not detected by novel biomarkers of AKI

**Fig 9:**
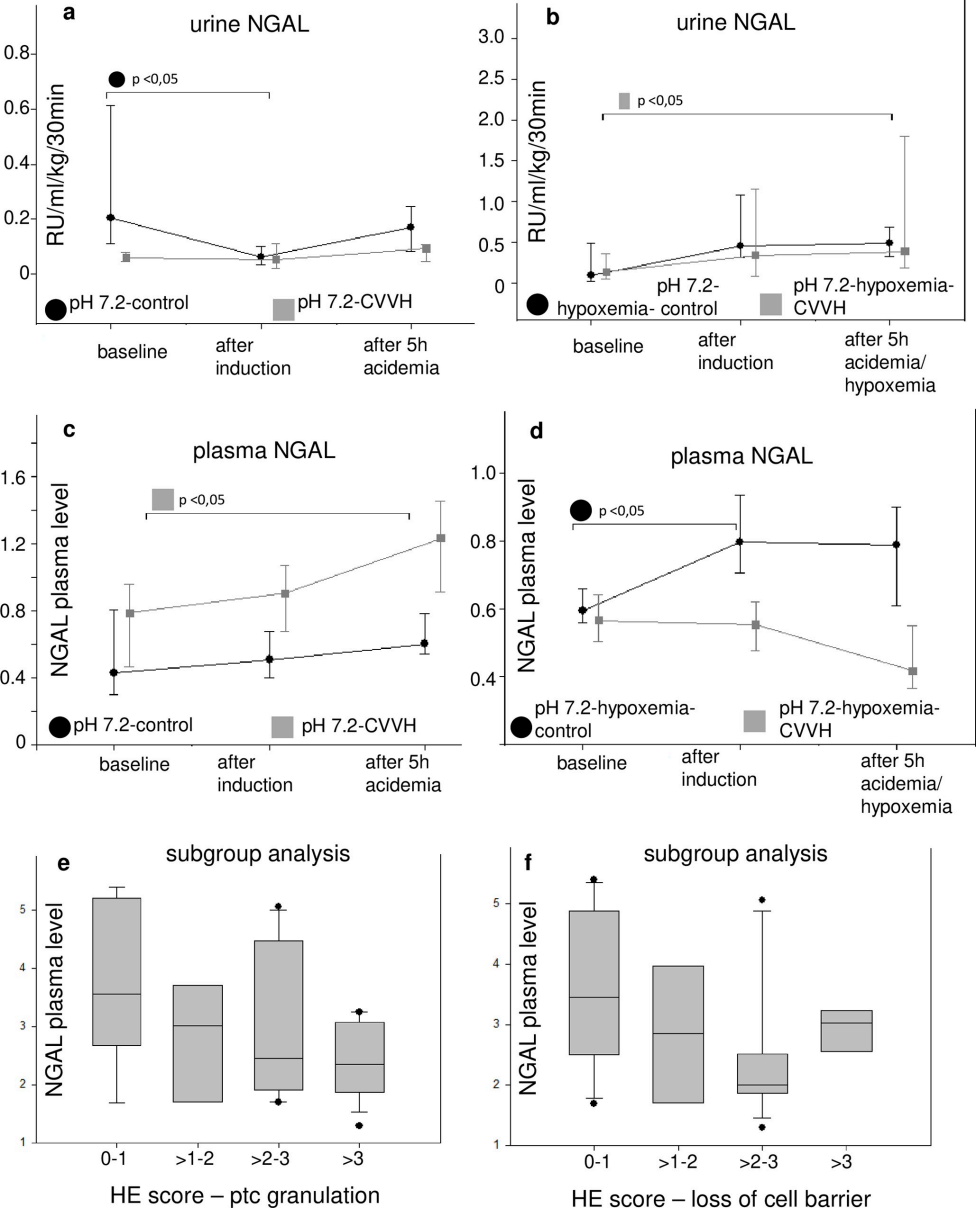
Histopathology, urine NGAL and plasma NGAL. The serum or urine concentrations of neutrophil gelatin-associated lipocalin (NGAL) are depicted in a-d. The values are presented as scatter plots (median/25th/75th percentile). The respective subgroup analysis (e, f) compare pooled subgroups for the semiquantative score for proximal tubular cell (PTC) granulation (a) and loss of cell barrier (b) in hematoxylin eosin (HE) stained slices at 400:1 enlargement with serum NGAL concentrations. The abbreviations pH 7.4-control. etc. mark the experimental groups as described in the article. Bracelets mark p < 0.0.5.

Although, urine neutrophil gelatin-associated lipocalin (NGAL) concentrations increased in the acidemic/hypoxemic CVVH-group, an irregular pattern was observed with respect to all groups as well as urine and serum NGAL concentrations after exposure to acidemia for five hours (Table 1, Fig 9). Accordingly, there was no significant increase in urine IL-18 concentrations. Subgroup analysis of pooled groups with high vs. low HE scores for kidney cell damage and serum NGAL concentrations did not indicate that a high degree of cell damage was mirrored by high NGAL concentrations (Fig 9).

## Discussions

Clinically, urinary output and laboratory measurements like serum creatinine are used to evaluate kidney function. Even immunological mediator levels, advanced hemodynamic monitoring and medical imaging can only give an indirect reflection of kidney perfusion, morphology and function. The hardest criterion of kidney cell damage is a biopsy and histopathological examination, which is almost never achievable in the critically ill patient. Thus, experimental investigations are necessary to identify conditions that result in cell damage and in turn AKI. To our knowledge, this was the first time that the impact of mixed acidemia on kidney function and histomorphology was investigated in large animals under clinical conditions.

Unexpectedly, we found a severe impact of acidemia itself on the kidneys’ morphological integrity after only three hours of exposure to arterial pH values of 7.2 (about five hours with induction of the pathology). The cell damages were observed despite normal to high diuresis rates and without a relevant increase of novel biomarkers of AKI. A causal relationship between kidney damage and ventilation is unlikely, since the observed kidney cell damage was less in the groups which were exposed to normal acid-base balance and simultaneously the highest inspiratory pressures and tidal volumes as compared to the acidemic groups with low tidal volume ventilation. Furthermore, Tsuno et al. ventilated piglets with a tidal volume of 13 ml/kg body weight for 48 hours and did not observe histopathological changes compared to lungs of healthy piglets [22].

An impact related to a reduced kidney perfusion was mainly excluded by using advanced hemodynamic monitoring. This was crucial since an impairment of systemic circulation can result in a reduction of renal blood flow (RBF), a decreased glomerular filtration rate (GFR) and a decreased renal clearance rates–regardless of the underlying pathomechanism. Direct measurements of RBF are hard to perform in critical ill patients and as a severe limitation we were not able to conduct direct measurements of RBF in this complex experimental setting. Nevertheless, it is known that even short warm ischemia due to cardiac arrest does not result in AKI [23] and experimentally reduced kidney perfusion of 80% for two hours did not result in AKI in sheep [24], whereas AKI occurred in experimental hyperdynamic sepsis in sheep despite an increased RBF [25, 26]. In the experiments presented here MAP was kept above 65 mmHg in the acidemic groups. Lower MAP values were accepted in the hypoxemic groups, which decompensated after an initial peak in MAP during the induction of hypoxemia. Nevertheless, cardiac output (CO) was always greater than 3 l/min at all times and the hemodynamic situation apparently enabled diuresis rates of at least 3 ml/kg/hr in all groups throughout the experiment.

An impact related to local or systemic inflammation was not likely since we did not use an animal model based on inflammation like a sepsis model. Furthermore, we did not find a distinct pattern of proinflammatory mediators–neither local nor systemic–which would support the hypothesis of an inflammatory response of the kidneys to an insult.

It is well known that the exposure of blood to an extracorporeal circuit triggers cascades of proinflammatory mediators [27]. On the other hand, operating CVVH in a high volume filtration mode can result in a partial hemofiltration of these molecules and additional adsorption of mediators always takes place [28–30]. However, we reinfused the filtrate into the venous bubble trap for the time of acidemia/hypoxemia which effectively prevented at least the hemofiltration of proinflammatory mediators during that time. Despite reinfusion of the filtrate, the acidemic as well as the acidemic/hypoxemic-control groups (no CVVH) had a higher increase of proinflammatory mediators in tendency than the CVVH-group with normal acid-base balance. This supports the hypothesis that the biotrauma caused by acidemia and the additional hypoxemia, respectively, was more serious than the exposure to the extracoporal circuit.

Nevertheless, it is difficult to interpret the relevance of proinflammatory mediators as long as organ function is not compromised. Novel biomarkers of AKI, such as IL-18 and NGAL, may detect AKI within a few hours of the incipient renal pathology, even before ‘traditionally’ used markers like serum creatinine or diuresis rate detect AKI [31, 32]. Interestingly, early histological cell damage was not indicated by a relevant increase in urine or plasma NGAL in the experiments presented here, as it can be observed e.g. in septic patients with AKI who can present with concentrations fourfold higher than non-septic patients [33].

Regardless of these findings, significantly more kidney cell damage was observed in the acidemic groups compared to groups without acidemia. The additional hypoxemia worsened the histopathological scores even further. It is well known that kidneys increase the excretion of acid equivalents during respiratory as well as metabolic acidosis. The kidneys’ response is ‘slow’ and three to five days pass before a new steady state is reached [34, 35]. Nevertheless, kidneys possess fast mechanisms to regulate hydrogen ion excretion. Especially active hydrogen ion-pumps (H^+^-ATPase) are the focus of recent research concerning acid-base regulation [36, 37]. Schwartz and Al-Awqati observed already in 1984 that H^+^-pump rich intracellular vesicles fuse with the luminal plasma membrane when the renal tubules of rabbits were exposed to an increased partial pressure of CO_2_ for just 15 minutes. Their work elegantly demonstrated that the major effect happened within the first minute of exposure and was accompanied by an increase in H^+^ secretion [38]. These processes are energy consuming–as all active ion-pumps [15].

Thus, the data presented here support the following hypothesis:

1. Kidneys are exposed to an energy consuming compensatory workload to regulate acid base-balance in case of acidemia, when regulation by the respiratory system is impaired
2. Cell damage will appear at an early stage–within hours–before clinically used markers detect AKI
3. This etiology may work as a ‘first hit’-insult potentiating further insults from ongoing biotrauma due to ventilation, impaired perfusion and inflammation in critically ill patients.

This hypothesis is supported by clinical data from a retrospective pooled analysis of three ARDS trials by Nin et al. Hypercapnia, defined as a maximum P_a_CO_2_ above 50 mmHg within the first 48 hours of mechanical ventilation, was associated with an increased ICU-mortality as well as a higher incidence of AKI in their work [39].

Furthermore, Jaber et al. demonstrated, that critically ill patients with a preexisting chronic kidney disease (Acute Kidney Injury Network score of 2 to 3 at study enrollment) profit from buffering a metabolic acidemia (defined as pH values below 7.2) with sodium bicarbonate with respect to days alive and days without RRT in a recent RCT [40].

This study has several limitations. In order to assess the possible impact of acidemia/hypoxemia on kidney function, the experiments were performed in healthy pigs without preexisting diseases and the experimental time was limited to seven hours. Contrary to the clinical situation, the experimental setting supported the harvest of tissue samples. However, tissue samples just reveal morphological and immunohistochemical changes at the time point of sampling. Furthermore, in our experiments kidney slices were scored for reversible markers of cell damage like cell granulation as well as irreversible changes like the loss of cell barriers. It remains unclear whether areas of the kidneys with potentially reversible changes would have developed irreversible changes over time. Some of the potentially reversible changes may present a physiological response to the energy consuming insult rather than a pathophysiological finding. Nevertheless, some cell changes like the loss of cell barrier are a permanent destruction of the tubules’ anatomic units.

At present, it is speculative to what extent the histological findings correlate with a clinically relevant compromised kidney function. Existing data suggests that healthy kidneys will sustain a considered amount of damage before kidney function is compromised at a clinical relevant level. Further research is needed to evaluate the role of acidemia as ‘first insult’ or contribution factor to AKI in a multifactorial setting.

## Conclusions

An exposure to a mixed acidemia of 5 hours resulted in histomorphological changes like a tubular loss of cell barriers in the kidneys of anesthetized healthy pigs. An exposure to an additional hypoxemia aggravated these histomorphological changes. The experimental model did not result in a systemic or local inflammation which could be correlated to histomorphology or kidney function. Kidney function as well as systemic perfusion were not compromised in this experimental setup. Instead, the additional energy-depending secretion of protons through the kidneys may have caused the histomorphological changes. Further investigations are needed about the exact pathomechanism and its possible relevance in critically ill patients.

## Supporting information

S1 Methods (DOCX)

S1 Fig Transmission electron microscopic pictures of renal tubular cells. The kidney slices were obtained in experiments investigating possible biocompatibiltiy reactions between continuous veno-venous hemofiltration (CVVH) and infusion of different colloids^1^; results and pictures concerning transmission electron microscopy not published). For this, anesthized pigs were exposed to CVVH and infusion of either 4% gelatine 30 kDa (Fig 6A and 6B) or 10% hydroxyethyl starch 200 kDa/0.5 (Fig c), but without exposure to acidemia. Ultra-thin slices (40–50 nm) were photographed at 1600:1 enlargement (Fig 6A and 6C) and 3150:1 enlargement (Fig b). BM marks the basal membrane, M marks a mitochondria, N marks the cell nucleus, ICV marks an intracellular vacuole. Note, multiple vacuoles of about 2,5 μm can be found in the tubular cells of the animal infused with either gelatine (6a, b) or starch (6c). Hence, relevant vacuolization due to starch infusion alone is not likely when compared to gelatine infusion.
(TIF)

S2 Fig Exemplary photos of the histomorphological criteria of kidney cell damage. Kidney slices were stained in hematoxylin eosin (HE) and photographed at 400:1 enlargement. A shows a slice with little cell damage, arrows indicate proximal tubular cell vacuolization. B and C are exemplary for high tubular cell damage with nucleus pycnosis (B) and cell barrier loss (C) highlighted with arrows. D shows normal morphology in comparison. * renal corpuscles, # proximal tubules, + distal tubules.
(TIF)

S3 Fig Exemplary photos of kidney slices at 200:1 enlargement stained with immunhistochemical staining (IHC) for IL-6; TNF-alpha or IL-18. All stained biomarkers occur in a brown colour as indicated by an arrow. The intensity of IHC staining was compared using as semiquantative score ranging from no IHC staining visible (0) to staining visible in more than 50% of the visual field (4).
A) Intensity of the signal score 1.B) Intensity of the signal score 2.C) Intensity of the signal score 3.D) Intensity of the signal score 4.E) Intensity of the signal score 0.
(TIF)

S1 Table Anesthetic drugs, volume management, devices, cannulation, induction of academia, hemofiltration. (DOCX)

S2 Table HE histopathology criteria. Scoring criteria for analyzing kidney cell damage in haematoxylin-eosin (HE) staining.
(DOCX)

S3 Table HE scoring values. The table summarizes which change in percent of a visual field for a certain score criteria equals which score value.
(DOCX)

S4 Table Materials for immunohistochemical (IHC) staining of kidney slices. (DOCX)

S5 Table IHC staining evaluated structures. The table summarizes the renal structures which were analyzed for immunohistochemical (IHC) staining.
(DOCX)

S6 Table Immunohistochemical (IHC) scoring values. The table provides details what qualitative IHC staining intensity equals which score value.
(DOCX)

S7 Table Materials for ELISAs. (DOCX)
