# Supplementary material for: Increased compensatory kidney workload results in cellular damage in a short time porcine model of mixed acidemia – Is acidemia a ‘first hit’ in acute kidney injury?
Source: PLoS One. 2019 Jun 17;14(6):e0218308. doi: 10.1371/journal.pone.0218308 (PMC6576776; doi:10.1371/journal.pone.0218308)
Supplement: S1 Methods — (DOCX) [file pone.0218308.s001.docx]

**S 1 Methods**

**Study groups**

The animals (male pigs, German landrace, large white, weight of 37-42 kg) were assigned to five groups (n = 6 all groups, except the historic control group with five animals respectively, see Fig 1). Two groups were exposed to a mixed acidemia, one with a continuous veno-venous hemofiltration (CVVH) and one without CVVH (control-group) under normoxic conditions (normoxic, acidemic groups). In two further groups (with and without CVVH) hypoxemia was additionally induced to acidemia (hypoxemic, acidemic groups). Another CVVH-group was exposed to normoxemia and a physiological acid-base balance (normoxemic CVVH-group with normal acid-base balance). Due to ethical aspects and strict local animal protection laws no new experiments were performed in a normoxemic control group (no CVVH) with normal acid-base balance, since these experiments were part of a former study [1]. Thus, only qualitative comparisons and no statistical tests were performed between the normoxemic control group with normal acid base balance and all other study groups

The detailed protocols of the porcine model of mixed (respiratory and metabolic) acidemia as well as hypoxemia/acidemia were reported previously [2, 3].

**General study design, anaesthesia, and fluid management**

After intramuscular premedication and induction of general anaesthesia the pigs were intubated (endotracheal tube, ID 7.0). Anaesthesia was continued as a total intravenous anaesthesia (TIVA, for details see supp. table 1). The body temperature was kept within a normal range of young pigs (38.0-39.0 °C). Initially, the pigs were normoventilated in with an end-tidal CO_2_ (etCO_2_) target of 35-40 mmHg and a saturation of peripheral oxygen (SpO_2_) above 95%, positive endexpiratory pressure (Peep) was set at 5 mmHg. The animals received an infusion of 4 ml/kg/h of 6% hydroxyethylstarch (HES) 130 kDa/0.4 % and an acetate-balanced crystalloid solution (Jonosteril acetat ®) to ensure hemodynamic stability during the period of acidemia/hypoxemia. The median HES dosage was 31 ml/kg with a crystalloid/colloid-ratio of 4:1. The fluid protocol originated from an experimental study concerning the biocompatibility of CVVH during colloid/crystalloid infusion in pigs.[1] In this study low HES with molecular weight or albumin yielded best results with respect to diuresis and stable hemodynamics compared to 0.9% saline, HES with a higher molecular weight or gelatin. Light microscopic as well as transmission electron microscopic evaluation supported the use of albumin or a low molecular weight HES in a short time experimental setting in pigs (S1 Fig). Due to cost effectiveness low molecular weight HES instead of albumin was used in the experiments presented here.

It has to be noted, that large clinical trials resulted in a higher incidence of kidney failure in critically ill patients who received starch-based colloids compared to crystalloids alone [4-6] and it has become clinical practice to avoid colloids in critically ill patients except albumin in septic shock or liver cirrhosis. Nevertheless, a continuous, fixed infusion of a balanced crystalloid and a colloid containing 6% hydroxyethyl starch was used for volume/fluid management in the experiments presented here. All animals received an equal amount of the same colloid and crystalloid. Hence, it is highly improbable that the infusion of HES caused the difference in kidney histomorphology between animals exposed to acidemia or acidemia/hypoxemia and animals exposed to an arterial pH of 7.4 and normoxemia.

**Cannulation**

After intubation the animals were placed in a supine position and cannulated after a cut down procedure with an arterial line, central venous line, shaldon catheter (for CVVH), introducer sheath and pulmonary arterial catheter (PAC). The urinary bladder was directly catheterized with a balloon catheter through a short paramedian laparotomy. Standard suture techniques were used to close the abdomen.

Following instrumentation measurements for baseline values were performed. Afterwards, acidemia or acidemia/hypoxemia was induced and the pigs were connected to the CVVH-circuit (not the control groups). The CVVH was operated for three hours by recirculation of the filtrate into the venous bubble trap while acidemia or acidemia/hypoxemia was maintained in the respective groups. The recirculation of the filtrate is based on a former study [1] and promotes possible hemocompatibility reactions towards the extracorporeal circuit, because inflammatory mediators are not removed with the filtrate. Anticoagulation was performed with continuous intravenous infusion of unfractionated heparin. The activated clotting time (ACT) was adjusted to 150–200 s. The total exposure to acidemia or acidemia/hypoxemia lasted 5 hours due to induction of the respective disorder for about 2 hours followed by 3 hours of ‘stable’ acidemia or acidemia/hypoxemia (Fig 1).

After 3 hours of stable acidemia or acidemia/hypoxemia the acid-base disorder was corrected within one hour by predilutional (before the hemofilter, Fig 2) infusion of 8 mmol/kg/hr of tris-hydroxymethylaminomethane-buffer (THAM) in the CVVH-groups or central-venous infusion of THAM (2mmol/kg/hr) in the control groups. CVVH-groups and control-groups received a different dosage of THAM due to the partial immediate filtration of THAM in the CVVH-animals. Hypoxemia was corrected during this ‘treatment’ period by adjusting F_I_O_2_ to 100%. Thus, final histopathology was performed after treatment of the underlying acidemia/hypoxemia.

**Acidaemia and hypoxemia protocol**

Target values for mixed acidemia were a pH of 7.19–7.24 and an arterial partial pressure of CO_2_ (P_a_CO_2_) of 80–85 mmHg. The additional target value for hypoxemia was a P_a_O_2_ below 70 mmHg and mixed venous oxygen saturation (S_v_O_2_) below 65%. Mixed acidemia was achieved by infusion of an acid solution (0.2 M lactic acid and 0.2 M hyperchloremic acid) and ‘low’ tidal volume ventilation (6 ml/kg). The acid infusion rate had to be adjusted individually in the hypoxemia-groups to reach the target range because the hypoxemia induced a metabolic acidosis itself.

The combination of an infusion of a mixture of lactic and hyperchloremic acid and low tidal volume ventilation (LTVV) enabled a time efficient induction of acidemia with comparable derangements in acid-base balance. The combination of acid infusion and LTTV was needed to induce hypercapnia since tidal volume ventilation with 6 ml/kg does not necessarily result in hypercapnia in pigs with healthy lungs. The infusion of lactic acid alone did not result in relevant acidemia in previous experiments to establish the animal model, most likely due to the high capacity of the healthy porcine livers to metabolize lactate [2].

The additional hypoxemia was induced by a stepwise reduction of the inspiratory oxygen fraction (F_I_O_2_). The reduction of the fraction of inspired oxygen (F_I_O_2_) %) to a fraction of about 0.3 was sufficient to induce hypoxemia (P_a_O_2_ < 70 mmHg, S_v_O_2_ < 65%) [3]. This indicates lung injury, since ventilation with air or fractions of oxygen above 0.21 should not result in hypoxemia in case of healthy lungs. Lung injury was most likely caused by acid infusion [7] and prolonged ventilation of the pigs in supine position which promotes atelectasis formation. The acid-base disorder was maintained for only three hours while a continuous veno-venous hemofiltration (CVVH) was started and then corrected by infusion of tris-hydroxymethylaminomethane (THAM) as buffer. Groups without CVVH served as control (Fig. 1).

**Laboratory analyses and hemodynamic measurements**

All samples for blood analyses were drawn from the central venous catheter (or PAC for SvO_2_) and immediately processed. Blood samples were analyzed at the local Institute for Clinical Chemistry, Charité - Universitätsmedizin Berlin using laboratory standard methods except for blood gas samples (ABL700; Radiometer, Copenhagen, Denmark). Serum and urine levels of interleukin 18 (IL-18), interleukin 6 (IL-6) and tumor necrosis factor alpha (TNF-α) were determined by enzyme linked immunoabsorbant assay (ELISA).

Hemodynamic measurements were performed with a Hewlett Packard monitor (HP 66S®; Bad Homurg, Germany) and ventilation was monitored with the ventilator (Ventilator 711, Siemens, Forchheim, Germany). Cardiac output (CO) was measured with the thermodilution technique and pulmonary artery occlusion pressure (PAOP) as determinant for left ventricular preload was measured at all main time points of the experiment using the PAC. Furthermore, oxygen delivery (DO_2_) and oxygen consumption (VO_2_) were calculated using standard equations [8].

We investigated histopathological scores of the kidneys, and mediator levels (IL-6, TNF-α, IL-18 in plasma, tissue and urine) besides monitoring of intra vitam renal function with diuresis and clearance as well as acid base balance of blood and urine. Finally, we measured urinary and plasma levels of NGAL and IL-18 concentrations which recently became a promising prognostic marker for acute kidney injury (AKI) [9-16]

**Histopathology**

After the experiment the pigs were euthanized by a bolus of fentanyl (0.5 mg), thiopentone (1 g) and potassium chloride (60 mmol) and the kidneys were immediately removed and small corticomedular tissue samples were fixated in a 4 vol% buffered formalin solution. The no-flow time of the kidneys between cardiac arrest and fixation were about ten minutes. After the fixation process tissue samples were dehydrated by a standard procedure of ascending alcohols and embedded in paraffin afterwards. Tissue slides of 4 µm thickness were cut. Afterwards the tissue slides underwent a standard haematoxylin-eosin (HE) staining by a standardised staining protocol.

**Hematoxylin-eosin (HE)-score criteria and gradation**

To evaluate the histopathological changes in the kidney tissue samples a histopathological score was established by adjusting existing scoring systems [17-19] to our investigation. Supplementary table 2 displays the investigated scoring criteria and supplementary table 3 the gradation of the score. Photos of exemplary slices are provided in figure 4 and supplementary figure 2. Every tissue slice was evaluated using a visual field enlargement of 25:1 to get a first overview and was reevaluated and finally scored in a second step using a visual field enlargement of 400:1. Multiple visual fields were evaluated for both kidneys of each animal. The median value of 10 visual fields of each kidney equals one score value. Tissue samples were scored for various histopathological criteria. Finally, the most expressive criteria were chosen for evaluation to differentiate clearly between histopathological changes due to the experiment and possible damage due to euthanasia, removal of the kidneys and preparation of the slides. Therefore, proximal tubular cell granulation – which can be interpreted as preceded but potential reversible disorder of cell integrity – and loss of cell barrier – as an irreversible sign for cell death – were chosen to correlate the histopathology of the kidneys with clinical kidney function.

**Immunhistochemistry (IHC) staining**

IHC staining was performed for IL-18, IL-6 and TNF-α, respectively. Antibody staining was performed using an ABC-staining kit. The staining procedures were performed in concordance with producer’s recommendations and are described subsequently. The optimal antibody dilution was determined in several protocol establishing steps. The slides were deparaffinized with descending alcohol concentrations and xylol. Endogenous peroxidase was blocked by 0.3% hydrogen peroxide. Afterwards, the slides were placed in a humidified chamber and incubated in 1.5% normal serum for 45 minutes at room temperature. The normal serum was provided by the animal in which the secondary antibody had been raised. Primary antibody dilutions were 1:50 combined with 1.5% normal serum. Afterwards, incubation with the biotin-conjugated secondary antibody and AB enzyme reagent according [manufacturer](http://www.dict.cc/englisch-deutsch/manufacturer.html)’s recommendations was performed. Finally, the slides were stained with a peroxidase substrate and counterstained with Mayer’s hematoxylin. The time of the peroxidase reaction was standardized to reach comparable results. After this staining procedure the slices were immediately washed with several changes of distilled water and then rinsed in water for five minutes. Afterwards another dehydration procedure with descending alcohol concentrations and xylol was performed. Finally, a permanent mounting medium was added. Slices were washed three times with 1 x PBS between each step (see S4 Table)

The tissue samples were scored and imaged using a Leica DMRB (Leica Microsystems GmbH, Wetzlar, Germany) with an AxioCam MRc (Carl Zeiss MicroImaging GmbH, Jena, Germany) and AxioVision Rel. 4.7.2 (Carl Zeiss Imaging Solutions GmbH, Jena, Germany). The criteria of the IHC-score are provided by S5 Table and S6 Table. Photos of exemplary IHC stained slices are provided in Fig 6 and S3 Fig.

**ELISA Analysis**

Urine and plasma concentrations of neutrophil gelatinase-associated lipocalin (NGAL), IL-18, IL-6 and TNF-α were measured using ELISA analysis (S7 Table).

All ELISAs were performed as specified by the manufacturer’s standard protocols.

First, all plates were coated with the coating antibody for one night. Afterwards, the coating antibody was blocked for one hour at room temperature using an assay buffer. An associated standard curve was prepared according to a standard procedure of serial dilution with a concentrated protein for every plate. For NGAL no standard porcine protein was commercially available at the time of our investigation ELISA. In consequence, one certain urine sample was determined which served as a reference value on each plate to obtain comparable, relative values. Thus, urine concentrations are expressed as relative units (relative to the reference value) per ml urine per kg body weight of the pig per 30 min collecting time of urine (Ru/ml/kg/30min).

The plates were incubated with serum or urine samples respectively and combined with the corresponding detection antibody for two hours at room temperature. Afterwards the plates were incubated with streptavidin-HRP for 30 minutes and were finally developed by TMB-substrate. The substrate reaction was stopped after a definite time of five minutes. After stopping the TMB-reaction the plates were covered to be protected against room light until absorption measurement. Absorptions measurements were performed by Tecan SPECTRA Fluor Plus, MTX Lab. Systems, USA. Standard curves and associated concentrations of the samples were calculated by Magellan™ - Data Analysis Software, Tecan Group Ltd., Switzerland.

For every serum or urine sample multiple wells were performed to get an average value of multiple measurements. Between each step plates were washed four times using a wash buffer.

**Statistical analysis**

All data were analysed using Sigma Stat 3.1 for Windows. Non-parametrical tests were used, because some data failed normal distribution. Kruskal-Wallis One Way Analysis of Variance on Ranks was used to test for possible differences of the baseline values between groups. The intergroup comparison between two study groups was performed using the Mann-Whitney Rank Sum Test. Post hoc analysis (pairwise comparison) was done using either Tukey’s test for equal sample size or Dunn's method for an unequal sample size. Friedman Repeated Measures Analysis of Variance on Ranks was performed for intra-group comparisons. Statistical significance was assumed at p <0.05.

**References for S 1 Methods**

1. Unger JK, Haltern C, Kobela JB, Francis B, Rossaint R, Grosse-Siestrup C. Hydroxyethyl starch 130 kd/0.4 and albumin improve CVVH biocompatibility whereas gelatin and hydroxyethyl starch 200 kd/0.5 lead to adverse side effects of CVVH in anesthetized pigs. Shock. 2006;25(5):533-45.

2. Russ M, Esche V, Ott S, Bedarf J, Unger JK. Stable mixed acidemia in anesthetized pigs--a model for research on biocompatibility of hemofilters under a deteriorated acid-base balance. Artif Organs. 2011;35(2):192-6. PubMed PMID: 21323687.

3. Russ M, Ott S, Bedarf JR, Haacke N, Keckel T, Unger JK. Prolonged hypoxemia and acidemia in anesthetized pigs: a model for research on extracorporeal organ support in an intensive care setting. Int J Artif Organs. 2010;33(8):544-52. PubMed PMID: 20872349.

4. Brunkhorst FM, Engel C, Bloos F, Meier-Hellmann A, Ragaller M, Weiler N, et al. Intensive Insulin Therapy and Pentastarch Resuscitation in Severe Sepsis. NEJM. 2008;358(2):125-39.

5. Perner A, Haase N, Guttormsen AB, Tenhunen J KG, Åneman A, Madsen KR, et al. Hydroxyethyl Starch 130/0.42 versus Ringer's Acetate in Severe Sepsis. N Engl J Med. 2012 Jul.;367(2):124-34. doi: 10.1056/NEJMoa1204242. Epub 2012 Jun 27.

6. Myburgh JA, Finfer S, Bellomo R, Billot L, Cass A, Gattas D, et al. Hydroxyethyl starch or saline for fluid resuscitation in intensive care. N Engl J Med. 2012 Nov;367(20):1901-11. doi: 10.1056/NEJMoa1209759. Epub 2012 Oct 17.

7. Matute-Bello G, Frevert CW, TR. M. Animal models of acute lung injury. Am J Physiol Lung Cell Mol Physiol. 2008;295(3):379-99. doi: 10.1152/ajplung.00010.2008. Epub 2008 Jul 11.

8. Al-Salam Z, Johnson S, Abozaid S, Bigam D, Cheung PY. The Hemodynamic Effects of Dobutamine during Reoxygenation after Hypoxia: A Dose-Response Study in Newborn Pigs. Shock. 2007;28(3):317-25.

9. Parikh CR, Abraham E, Ancukiewicz M, Edelstein CL. Urine IL-18 is an early diagnostic marker for acute kidney injury and predicts mortality in the intensive care unit. J Am Soc Nephrol. 2005;16(10):3046-52. PubMed PMID: 16148039.

10. Parikh CR, Mishra J, Thiessen-Philbrook H, Dursun B, Ma Q, Kelly C, et al. Urinary IL-18 is an early predictive biomarker of acute kidney injury after cardiac surgery. Kidney Int. 2006;70(1):199-203. PubMed PMID: 16710348.

11. Parikh CR, P D. New biomarkers of acute kidney injury. Crit Care Med. 2008 Apr;36(4 Suppl):159-65. doi: 10.1097/CCM.0b013e318168c652.

12. Mehta RL. Urine IL-18 levels as a predictor of acute kidney injury in intensive care patients. Nat Clin Pract Nephrol. 2006;2(5):252-3. PubMed PMID: 16932437.

13. Coca SG, Yalavarthy R, Concato J, Parikh CR. Biomarkers for the diagnosis and risk stratification of acute kidney injury: a systematic review. Kidney Int. 2008;73(9):1008-16. PubMed PMID: 18094679.

14. Ronco C. N-GAL: diagnosing AKI as soon as possible. Crit Care. 2007;11(6):173.

15. Schmidt-Ott KM, Mori K, Kalandadze A, Li JY, Paragas N, Nicholas T, et al. Neutrophil gelatinase-associated lipocalin-mediated iron traffic in kidney epithelia. Curr Opin Nephrol Hypertens. 2006;15(4):442-9. PubMed PMID: 16775460.

16. Haase M, Bellomo R, Devarajan P, Schlattmann P, Haase-Fielitz A. Accuracy of neutrophil gelatinase-associated lipocalin (NGAL) in diagnosis and prognosis in acute kidney injury: a systematic review and meta-analysis. Am J Kidney Dis. 2009;54(6):1012-24. PubMed PMID: 19850388.

17. Dittrich S, Groneberg DA, von Loeper J, Lippek F, Hegemann O, Grosse-Siestrup C, et al. Influence of cold storage on renal ischemia reperfusion injury after non-heart-beating donor explantation. Nephron Exp Nephrol. 2004;96(3):e97-102. PubMed PMID: 15056986.

18. Torras J, Herrero-Fresneda I, Lloberas N, Riera M, Ma Cruzado J, Ma Grinyo J. Promising effects of ischemic preconditioning in renal transplantation. Kidney Int. 2002;61(6):2218-27. PubMed PMID: 12028463.

19. Jablonski P, Howden BO, Rae DA, Birrell CS, Marshall VC, Tange J. An experimental model for assessment of renal recovery from warm ischemia. Transplantation. 1983;35(3):198-204. PubMed PMID: 6340272.
