# Supplementary figures and images for: Increased compensatory kidney workload results in cellular damage in a short time porcine model of mixed acidemia – Is acidemia a ‘first hit’ in acute kidney injury?
Source: PLoS One. 2019 Jun 17;14(6):e0218308. doi: 10.1371/journal.pone.0218308 (PMC6576776; doi:10.1371/journal.pone.0218308)

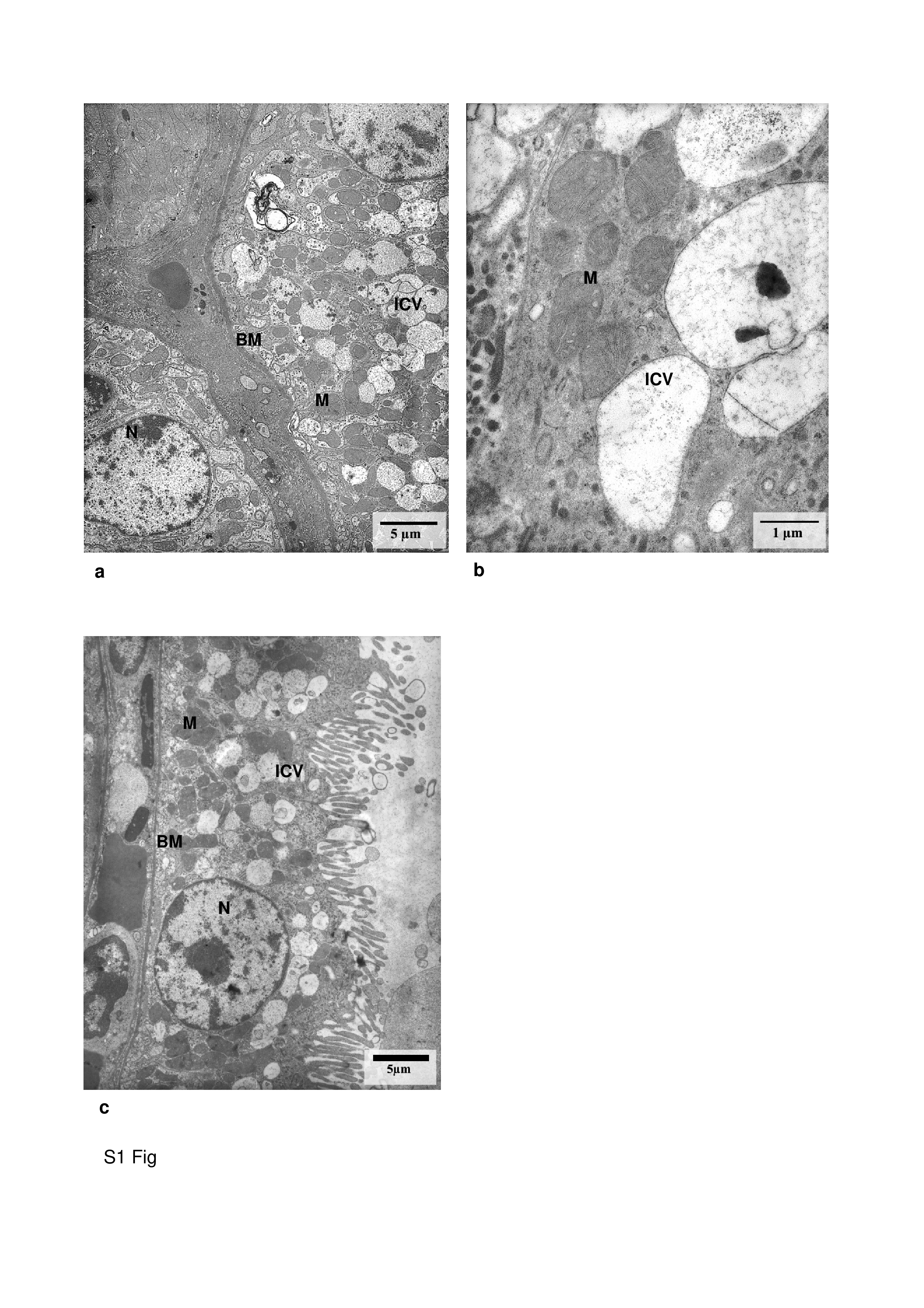

Supplement: S1 Fig — The kidney slices were obtained in experiments investigating possible biocompatibiltiy reactions between continuous veno-venous hemofiltration (CVVH) and infusion of different colloids1; results and pictures concerning transmission electron microscopy not published). For this, anesthized pigs were exposed to CVVH and infusion of either 4% gelatine 30 kDa (Fig 6A and 6B) or 10% hydroxyethyl starch 200 kDa/0.5 (Fig c), but without exposure to acidemia. Ultra-thin slices (40–50 nm) were photographed at 1600:1 enlargement (Fig 6A and 6C) and 3150:1 enlargement (Fig b). BM marks the basal membrane, M marks a mitochondria, N marks the cell nucleus, ICV marks an intracellular vacuole. Note, multiple vacuoles of about 2,5 μm can be found in the tubular cells of the animal infused with either gelatine (6a, b) or starch (6c). Hence, relevant vacuolization due to starch infusion alone is not likely when compared to gelatine infusion. (TIF) [file pone.0218308.s002.tif]

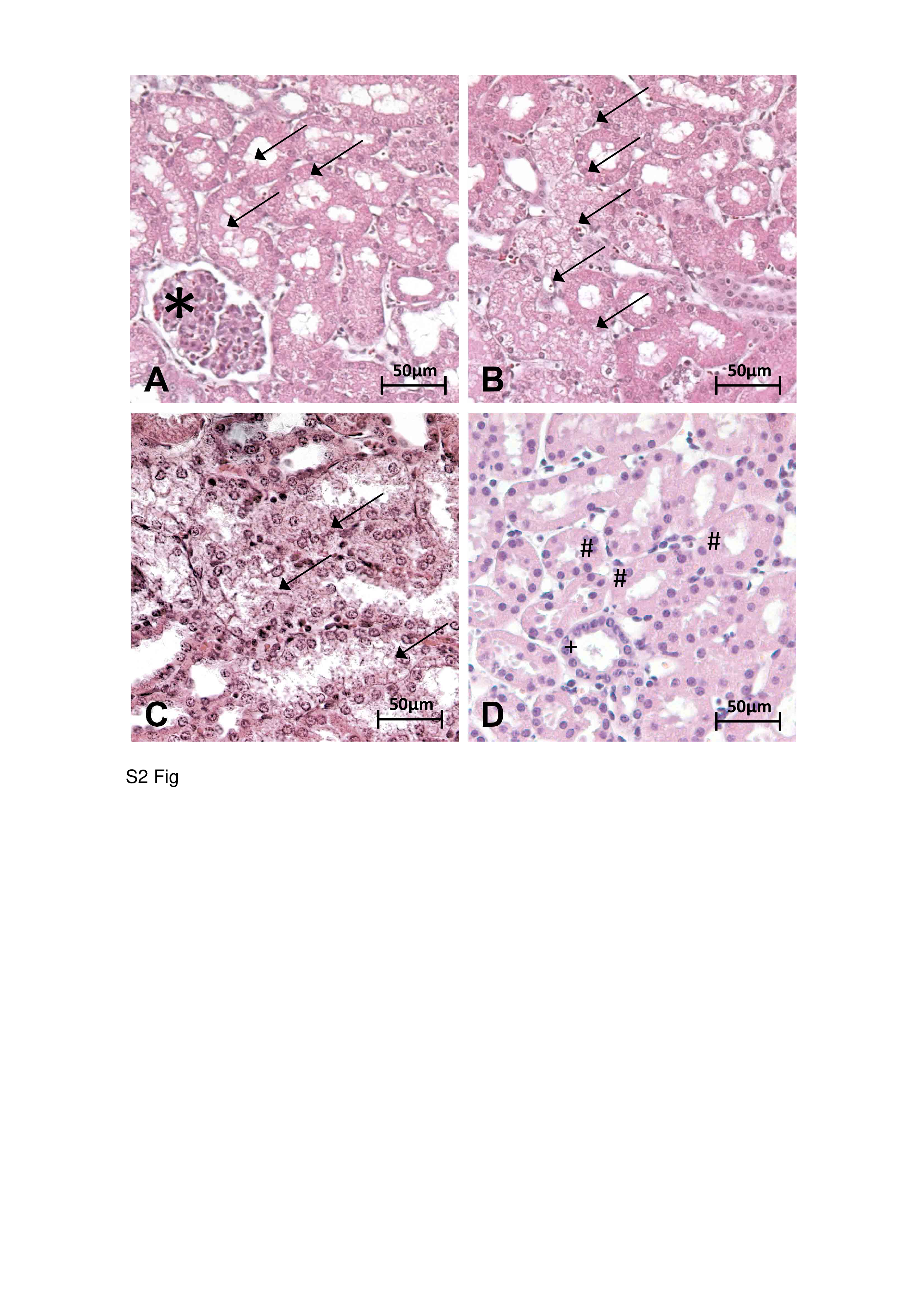

Supplement: S2 Fig — Kidney slices were stained in hematoxylin eosin (HE) and photographed at 400:1 enlargement. A shows a slice with little cell damage, arrows indicate proximal tubular cell vacuolization. B and C are exemplary for high tubular cell damage with nucleus pycnosis (B) and cell barrier loss (C) highlighted with arrows. D shows normal morphology in comparison. * renal corpuscles, # proximal tubules, + distal tubules. (TIF) [file pone.0218308.s003.tif]

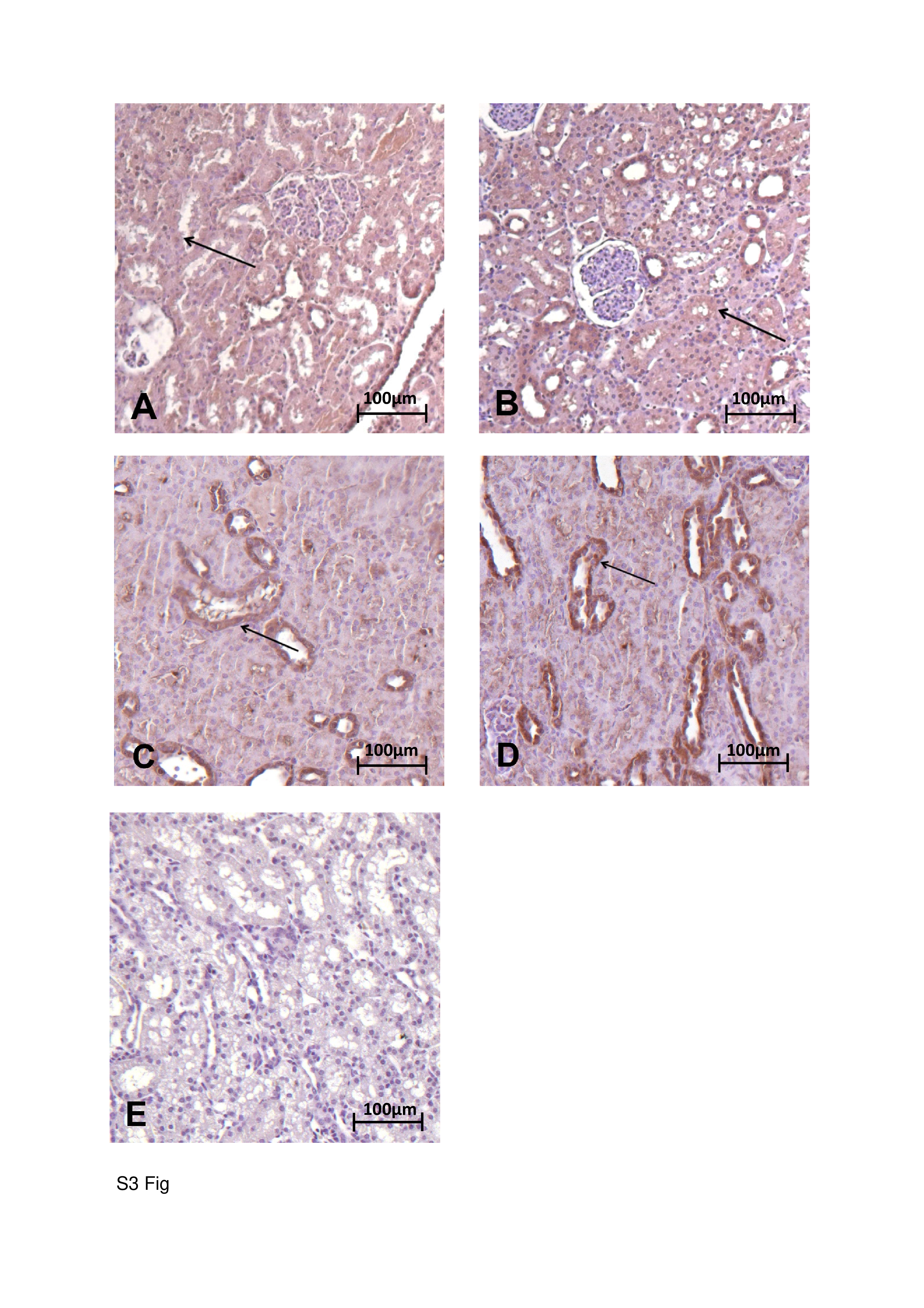

Supplement: S3 Fig — All stained biomarkers occur in a brown colour as indicated by an arrow. The intensity of IHC staining was compared using as semiquantative score ranging from no IHC staining visible (0) to staining visible in more than 50% of the visual field (4). A) Intensity of the signal score 1.B) Intensity of the signal score 2.C) Intensity of the signal score 3.D) Intensity of the signal score 4.E) Intensity of the signal score 0. (TIF) [file pone.0218308.s004.tif]
