# Supplementary material for: Increased compensatory kidney workload results in cellular damage in a short time porcine model of mixed acidemia – Is acidemia a ‘first hit’ in acute kidney injury?
Source: PLoS One. 2019 Jun 17;14(6):e0218308. doi: 10.1371/journal.pone.0218308 (PMC6576776; doi:10.1371/journal.pone.0218308)
Supplement: S1 Table — (DOCX) [file pone.0218308.s005.docx]

**S1 Table Anesthetic drugs, volume management, devices, cannulation, induction of academia, hemofiltration.**

| **Premedication, intramuscular application** | | | |
| --- | --- | --- | --- |
| azaperone: 3 mg·kg^-1^ (Stressnil®, Janssen-Cilag, Neuss, Germany) | | | |
| atropine sulphate: 0.03 mg·kg^-1^ (Atropin sulfat®, B. Braun Melsungen AG,  Melsungen, Germany) | | | |
| ketamine: 25 mg·kg^-1^ (Ursotamin®, Serumwerk, Bernberg, Germany) | | | |
| xylazine hydrochloride: 3.7 mg·kg^-1^ (Rompun®, Bayer Vital GmbH,  Leverkusen,Germany) | | | |
| **Total intravenous anaesthesia (TIVA)** | | | |
| bolus of propofol: 7-10 mg·kg^-1^ for intubation (Propofol 1% MCT®, Fresenius Kabi, Bad  Homburg, Germany) | | | |
| thiopental: 17.5-20 mg·kg^-1^·h^-1^  (Trapanal®, ALTANA Pharma, Konstanz, Germany) | | | |
| fentanyl: 5.5 µg·kg^-1^·h^-1^ (Fentanyl®, Janssen-Cilag, Neuss, both  Germany) | | | |
| **Volume management** | | | |
| jonosteril acetate® (Fresenius Kabi, Bad Homburg, Germany) during instrumentation: 24 ml·kg^-1^·h^-1^ | | | |
| hydroxyethylstarch 6% (HES) 130 kDa/0.4: initial bolus of 15 ml·kg^-1^, continuous infusion of 2.5 ml·kg^-1^·h^-1^ | | | |
| **Devices** | | | |
| ventilator: Ventilator 711® (Siemens, Germany) | | | |
| warming device: Warm Touch ^TM®^(Tyco Healthcare, Neustadt, Germany) | | | |
| ACT: Hemochron 400®, AD Krauth, Cardiovascular Vertriebsgesellschaft, Hamburg, Germany | | | |
| **Instrumentation** | | | |
|  | **vascular access** | **catheter** | **function** |
|  | left external jugular vein | 4-Fr central venous catheter (Arrow, Erdingen, Germany) | central venous access; acid infusion |
|  | right external jugular vein | 8.5-Fr sheath (Arrow, Erdingen, Germany)  pulmonary artery catheter  (Criti-Cath™; SP5127 S-TIP TD;  Becton Dickinson, Heidelberg, Germany) | hemodynamic monitoring |
|  | right femoral vein | double-lumen catheter (13 Fr, 20 cm length, tip-hole catheter; Gambro, Hechingen, Germany) | connection to the CVVH system |
|  | right femoral artery | arterial catheter (Combidyn®; B. Braun, Melsungen, Germany) | invasive blood pressure, blood gas analysis |
| **Acid infusion** | | | |
| acid solution: 0.2 M of lactic acid and 0.2 M hydrochloric acid, diluted in normal saline | | | |
| acidemic groups: bolus infusion of 1.8 mmol·kg^-1^ over 75min followed by continuous infusion of 2.1 mmol·kg^-1^·h^-1^ | | | |
| acidemic/hypoxemic-groups: bolus infusion of 1.8 mmol·kg^-1^ over 75min followed by an individually adjusted continuous infusion (mean of 0.6 mmol·kg^-1^·h^-1^) | | | |
| groups with normal acid-base balance: received an equal volume of normal saline to ensure an equal fluid status between the groups | | | |
| **Continuous venovenous haemofiltration** | | | |
| Gambro AK10® (Gambro Hospal GmbH, Gröbenzell, Germany) with medical-grade poly-vinyl-chloride (PVC) tubes and polyamide ST membrane filters (Polyflux 140H®) | | | |
| anticoagulation: unfractionated heparin (Liquemin®, Hoffmann-La Roche AG, Grenzach-Wyhlen, Germany), started with a bolus of 60 IU·kg^-1^, followed by an continuous infusion of 60 IU·kg^-1^·h^-1^ | | | |
