# Supplementary material for: Increased compensatory kidney workload results in cellular damage in a short time porcine model of mixed acidemia – Is acidemia a ‘first hit’ in acute kidney injury?
Source: PLoS One. 2019 Jun 17;14(6):e0218308. doi: 10.1371/journal.pone.0218308 (PMC6576776; doi:10.1371/journal.pone.0218308)
Supplement: S2 Table — Scoring criteria for analyzing kidney cell damage in haematoxylin-eosin (HE) staining. (DOCX) [file pone.0218308.s006.docx]

**S2 Table. HE histopathology criteria.**

| proximal tubular cell granulation (advanced signs of nucleus pycnosis and plasma changes) |
| --- |
| loss of cell barrier |
| proximal tubular cell vacuolation |
| brush border loss of proximal tubular cells |
| nucleus migration to the apical pole in proximal tubular cells |
| proximal tubular cell edema |
| dilatation of proximal tubulus diameter |
| peritubular and perivascular infiltration of leukocytic cells |
| interstitial edema |

Scoring criteria for analyzing kidney cell damage in haematoxylin-eosin (HE) staining.
