# Supplementary material for: Increased compensatory kidney workload results in cellular damage in a short time porcine model of mixed acidemia – Is acidemia a ‘first hit’ in acute kidney injury?
Source: PLoS One. 2019 Jun 17;14(6):e0218308. doi: 10.1371/journal.pone.0218308 (PMC6576776; doi:10.1371/journal.pone.0218308)
Supplement: S3 Table — The table summarizes which change in percent of a visual field for a certain score criteria equals which score value. (DOCX) [file pone.0218308.s007.docx]

**S3 Table. HE scoring values.**

| score value | Indicates the change in percent (%) of the visual field for the respective criteria |
| --- | --- |
| 0 | no change |
| 1 | <15% change |
| 2 | 16-30% change |
| 3 | 31-50% change |
| 4 | >50% change |
| for leukocytic cells the total amount per visual field was counted | |
| multiple visual fields were evaluated from each kidney, a median value of 10 visual fields of each kidney equals one score value | |

The table summarizes which change in percent of a visual field for a certain score criteria equals which score value.
