# Supplementary material for: Increased compensatory kidney workload results in cellular damage in a short time porcine model of mixed acidemia – Is acidemia a ‘first hit’ in acute kidney injury?
Source: PLoS One. 2019 Jun 17;14(6):e0218308. doi: 10.1371/journal.pone.0218308 (PMC6576776; doi:10.1371/journal.pone.0218308)
Supplement: S4 Table — (DOCX) [file pone.0218308.s008.docx]

**S4 Table. Materials for immunohistochemical (IHC) staining of kidney slices.**

| IL-6 | IL-6 (F20-1), sc-80106, santa cruz biotechnology inc., Santa Cruz, CA, USA |
| --- | --- |
| TNF alpha | TNF α (52B83), sc-52746, santa cruz biotechnology inc., Santa Cruz, CA, USA |
| IL-18 | IL-18 (N-19), sc-6178, santa cruz biotechnology inc., Santa Cruz, CA, USA |
| ABC-staining | goat-ABC staining system (sc-2023, santa cruz biotechnology inc., Santa Cruz, USA) for IL-18  mouse-ABC staining system (sc-2017, santa cruz biotechnology inc., Santa Cruz, USA) for IL-6 and TNF-aplha |
