# Supplementary material for: Increased compensatory kidney workload results in cellular damage in a short time porcine model of mixed acidemia – Is acidemia a ‘first hit’ in acute kidney injury?
Source: PLoS One. 2019 Jun 17;14(6):e0218308. doi: 10.1371/journal.pone.0218308 (PMC6576776; doi:10.1371/journal.pone.0218308)
Supplement: S5 Table — The table summarizes the renal structures which were analyzed for immunohistochemical (IHC) staining. (DOCX) [file pone.0218308.s009.docx]

**S5 Table. IHC staining evaluated structures.**

| proximal tubular cells |
| --- |
| distal tubular cells |
| cells of Henle’s loop |
| collecting duct cells |
| tubulus lumen |
| blood vessel endothelial cells |

The table summarizes the renal structures which were analyzed for immunohistochemical (IHC) staining.
