# Supplementary material for: Increased compensatory kidney workload results in cellular damage in a short time porcine model of mixed acidemia – Is acidemia a ‘first hit’ in acute kidney injury?
Source: PLoS One. 2019 Jun 17;14(6):e0218308. doi: 10.1371/journal.pone.0218308 (PMC6576776; doi:10.1371/journal.pone.0218308)
Supplement: S6 Table — The table provides details what qualitative IHC staining intensity equals which score value. (DOCX) [file pone.0218308.s010.docx]

**S6 Table. Immunohistochemical (IHC) scoring values.**

| score | indicates for… |
| --- | --- |
| 0 | no signal intensity |
| 1 | low signal intensity |
| 2 | medium signal intensity |
| 3 | high signal intensity |
| 4 | very high signal intensity |
| Multiple visual fields were evaluated from each kidney | |

The table provides details what qualitative IHC staining intensity equals which score value.
