# Supplementary material for: Increased compensatory kidney workload results in cellular damage in a short time porcine model of mixed acidemia – Is acidemia a ‘first hit’ in acute kidney injury?
Source: PLoS One. 2019 Jun 17;14(6):e0218308. doi: 10.1371/journal.pone.0218308 (PMC6576776; doi:10.1371/journal.pone.0218308)
Supplement: S7 Table — (DOCX) [file pone.0218308.s011.docx]

**S7 Table. Materials for ELISAs.**

| IL-6 | IL-6 Matched Antibody Pairs BMS213/2MST, Bender Medsystems GmbH, Austria |
| --- | --- |
| TNF alpha | Swine TNF- a CytoSet™ CSC1753, Invitrogen, Carlsbad, CA, USA |
| IL-18 | IL-18 Matched Antibody Pairs BMS672MST, Bender Medsystems GmbH, Austria |
| NGAL | coating antibody ABS 048-17-02, detecting antibody ABS 048-28B-005, Streptavidin HRP 016-030-084, all reagents from dianova, Germany |
